# Polysaccharide isolated from* Grifola frondosa* eliminates myeloid-derived suppressor cells and inhibits tumor growth by enhancing T cells responses

**DOI:** 10.7150/ijbs.85276

**Published:** 2024-01-01

**Authors:** Xiangmin Li, Qinzhao Ruan, Weining Yang, Huixiang Tian, Nan Wu, Javeria Qadir, Juan Wang, Huiping Hu, Yuanchao Liu, Manjun Cai, Burton B. Yang, Yizhen Xie, Qingping Wu

**Affiliations:** 1 State Key Laboratory of Applied Microbiology Southern China, Guangdong Provincial Key Laboratory of Microbial Safety and Health,National Health Commission Science and Technology Innovation Platform for Nutrition and Safety of Microbial Food, Institute of Microbiology, Guangdong Academy of Sciences, Guangzhou 510070, China.; 2 College of Food Science, South China Agricultural University, Guangzhou 510642, China.; 3 Sunnybrook Research Institute, Sunnybrook Health Sciences Centre, Toronto, M4N3M5, Canada.; 4 Department of Laboratory Medicine and Pathobiology, University of Toronto, Toronto, M4N3M5, Canada.; 5 Yuewei Edible Fungi Technology Co. Ltd., Guangzhou 510070, China.

**Keywords:** *Grifola frondosa*, polysaccharide, breast cancer, Myeloid derived suppressor cells, CD8^+^T cells

## Abstract

Myeloid derived suppressor cells (MDSCs) are known to accumulate in cancer patients and tumor-bearing mice, playing a significant role in promoting tumor growth. Depleting MDSCs has emerged as a potential therapeutic strategy for cancer. Here, we demonstrated that a fungal polysaccharide, extracted from *Grifola frondosa,* can effectively suppress breast tumorigenesis in mice by reducing the accumulation of MDSCs. Treatment with *Grifola frondosa* polysaccharide (GFI) leads to a substantial decrease in MDSCs in the blood and tumor tissue, and a potent inhibition of tumor growth. GFI treatment significantly reduces the number and proportion of MDSCs in the spleen, although this effect is not observed in the bone marrow. Further analysis reveals that GFI treatment primarily targets PMN-MDSCs, sparing M-MDSCs. Our research also highlights that GFI treatment has the dual effect of restoring and activating CD8^+^T cells, achieved through the downregulation of TIGIT expression and the upregulation of Granzyme B. Taken together, our findings suggest that GFI treatment effectively eliminates PMN-MDSCs in the spleen, leading to a reduction in MDSC numbers in circulation and tumor tissues, ultimately enhancing the antitumor immune response of CD8^+^T cells and inhibiting tumor growth. This study introduces a promising therapeutic agent for breast cancer.

## Introduction

Myeloid-derived suppressor cells (MDSCs) are a highly heterogenic populations of immature myeloid cells with immunosuppressive effects in cancers^1^, ^2^. MDSCs are massively expanded in the cancer patients and tumor-bearing mice, and the density of MDSCs in the circulatory system and tumor sites is positively correlated with the tumor burden but negatively correlated with shorter survival and less sensitivity to antitumoral therapy in different types of cancer, such as breast cancer, prostate cancer, colorectal cancer, and lung adenocarcinoma^3^-^6^. MDSCs include two subsets, polymorphonuclear (PMN)-MDSCs and monocytic (M)-MDSCs, based on cells morphological and phenotypic characteristics^1^, ^7^. Both cell populations can inhibit tumoricidal immune cells to promote immune escape of tumor cells and tumor growth, using different effector molecules and signaling pathways 8, 9. More findings indicated that MDSCs are one of the major immunosuppressive factors in the tumor environment of malignant tumors, and are also potential targets for cancer therapy. Therefore, reducing MDSCs density in tumor-bearing host could act as an effective cancer immunotherapy approach.

Currently, it has been reported that multiple conventional drugs, such as 5-fluorouracil, docetaxel, gemcitabine, and chemokine inhibitors, depleted MDSCs and consequently improved the efficacy of tumor immunotherapy in the tumor-bearing host^2^, ^6^, ^10^, ^11^. However, these agents were not specific for MDSCs and may affect T cell proliferation 10-12. Some natural products targeting MDSCs, such as curcumin and β‐Glucan from yeast, have been investigated in preclinical and clinical studies^2^, ^13^. In our previous studies, more than ten medicinal fungi extract for the antitumor effect were screened using mouse 4T1 breast tumor model, among which polysaccharide, extracted from *Grifola frondosa*, significantly inhibited tumor growth and prevented spleen enlargement (Supplementary Fig. S1). Some studies have shown that splenomegaly in 4T1 mammary carcinoma bearing mice was caused by the expansion and accumulation of MDSCs^14^, ^15^. *Grifola frondosa* is a fungus of polyporaceae family, which is widely used in East Asia as a medicinal and edible mushroom. The main bioactive compounds of *G. frondosa* are polysaccharide^16^-^18^. It has been found that polysaccharide and polysaccharide-protein complex from* G. frondosa* suppressed tumor growth via improved immunity by enhancing the macrophage activity, controlling Th-1/Th-2 proportion, improving cytotoxic T-cells and natural killer (NK) cells, and decreasing the secretion of cytokines such as COX-2, IL-6 and TNF-α^17^, ^19^-^21^. Therefore, we hypothesized that GFI, polysaccharide-rich extract, could inhibit the expansion of MDSCs in mice, and enhance antitumor immune response leading to blockade of tumor growth.

In this study, we used triple-negative breast cancer cell models to study the antitumor effect of fungal polysaccharide via eliminating MDSCs and activating T cell responses. The 4T1 tumor-bearing mice have several characteristics in common with the human breast carcinomas^22^, ^23^. In 4T1 tumor-bearing mice, MDSCs accumulate at the tumor site, peripheral blood, spleen, and bone marrow^24^. Using this model, we evaluated the antitumor effect of GFI *in vitro* and *in vivo*, and found an immunological antitumor program mediating the effects of GFI in TNBC therapies. We demonstrated that GFI treatment significantly decreased the accumulation of MDSCs in the spleen, blood and tumor site, while it could not inhibit the expansion of MDSCs in the bone marrow in 4T1 tumor-bearing BALB/c mice. We further discovered that GFI treatment mainly decreased PMN-MDSCs, but not M-MDSCs. Furthermore, GFI treatment increased the proportion and activation of T cells in the tumor-bearing mice. GFI treatment also reduced the mRNA and protein expression of TIGIT, and increased the expression of granzyme B to restore and activate T cell response in the tumor micro-environment. Therefore, GFI, polysaccharide-rich extract from *G. frondosa*, enhanced host immunity to cancer by eliminating myeloid-derived suppressor cells.

## Materials and Methods

### Materials

The fruit bodies of *G. frondosa* were obtained from Guangdong Yuewei Edible Fungi Technology Co. Ltd., Guangzhou, China. Fetal bovine serum, goat serum, DMEM, PBS and penicillin/ streptomycin antibiotics were purchased from Thermo Fisher Scientific. HPLC grade sodium chloride (NaCl) was purchased from Sigma-Aldrich. Deionized water was purified by the Millipore-Q water purification system. All the antibody related information is listed in the supplementary Table S1. Horseradish peroxidase-conjugated goat anti-rabbit IgG was obtained from Cell Signaling Technology.

### Cell line and Mice

The murine 4T1 cells were purchased from ATCC. 4T1 breast cancer cells were cultured in DMEM medium supplemented with 10% fetal bovine serum, 1% Penicillin/ streptomycin antibiotics at 37°C in an incubator containing 5% CO_2_.

All animal experiments were conducted in accordance with a protocol approved by the Animal Core Facility of Institute of Microbiology, Guangdong Academy of Sciences. Five- to seven-week-old female BALB/c mice were purchased from Guangdong medical laboratory animal center. All the mice were housed under standard and specific pathogen-free conditions in the animal core facility of Institute of Microbiology, Guangdong Academy of Sciences.

### *Grifola frondosa* polysaccharide's information

*Grifola frondosa* polysaccharide (GFI) was isolated from the fruit bodies of *G. frondosa*. In brief, the dry fruit bodies of *G. frondosa* were ground to powder. The powder was soaked in deionized water at a ratio of 1:20 (w/v) for 30 minutes, and then extracted by refluxing two times at 100°C. The extract was filtered and concentrated to a small volume. The polysaccharide fraction was precipitated overnight at 4°C by adding 4 volumes of ethanol in the extract. The precipitate was re-dissolved in water, dialyzed against water for 72 hours, concentrated, filtered and dried by a vacuum freeze-drying.

The molar mass distribution of GFI was determined by high-performance gel filtration chromatography (HPGFC) on an Agilent 1200 high-performance liquid chromatography (HPLC) system with differential refraction detector (1260 Infinity, Agilent, USA). After filtered with 0.22 μm filter, the polysaccharide solution (5 mg/mL, 10 μL) was separated in the TSKgel G3000PWXL and TSKgel G5000PWXL columns (7.8 mm × 30 cm, Tosoh Bioscience, Tokyo, Japan), with 0.01 M NaCl elution solution at 0.5 mL/min at 35 °C. The average molecular weight of the polysaccharide ranged from 0.24 to 99.9 x 10^4^Da (Fig. S2 and Table S2).

### Lentinan isolation

Lentinan (LNT) was isolated from the fruit bodies of *Lentinula edodes*. Lentinan was extracted by water abstraction and alcohol precipitation as described under '*Grifola frondosa* polysaccharide's information' section.

### Mouse tumor model and treatments

On day 0 of the experiments, 0.5 x 10^5^ 4T1 cells were implanted subcutaneously. Animals were randomized on day 7 post injection and given the treatments with the following regimen for GFI. GFI in 0.9% saline were delivered intraperitoneally every other day until the end-point, with a dose of 25 mg/kg, 50 mg/kg and 100 mg/kg (100 μL at the concentrations prepared above). The control animals were treated with an equal volume of 0.9% saline. In the Supplementary Fig. S1, GFI (100 mg/kg, 100 μL) treatment was initiated on day 1 post tumor implantation and continued every other day until the end-point. To compare the *in vivo* activity of polysaccharides derived from GFI and LEN, mice were intraperitoneally administered GFI (50 mg/kg), LEN (50 mg/kg), or a control solution of 0.9% saline seven days after tumor cell injection. Tumor size was measured with a caliper on day 7, 14, 21, 28, and 32. Tumor volume was calculated using the formula (width^2^ × length)/2. At the end-point, the mice were sacrificed, and the tumors were removed, weighed and photographed. Tumor inhibitory rates were calculated using the formula (‾x-x)/ x*100, where '‾x' is the average tumor weight of the control group and 'x' is the tumor weight of each mouse in the treatment group. The doses of GFI (25 mg/kg, 50 mg/kg and 100 mg/kg) were represented with GFI-LD, GFI-MD and GFI-HD. The dose of Lentinan (50 mg/kg) was represented with LNT-MD.

### Cell proliferation, cell viability, cell apoptosis and cell cycle progression *in vitro*

Cell proliferation assay was used to evaluate the antitumor effect of *G. frondosa* polysaccharides (GFI) *in vitro* using trypan blue staining as previously described 25, 26. In brief, murine breast cancer cells 4T1 and human breast cancer cells MDA-MB-231were used in the study. The cells (1x10^5^ cells/mL, 0.5 mL/well) were seeded into 24-well cell plates and cultured in DMEM (containing 10% FBS and 100 U/mL penicillin/streptomycin) in an incubator containing 5% CO_2_ at 37°C. Four hours after cell inoculation, GFI was added to the cultures at different concentrations and incubated for 48 hours. Cell proliferation was analyzed by trypan blue staining. Each experiment was repeated for at least three times for statistical analysis.

Cell viability was assessed using a CCK-8 assay^27^. In brief, cells were seeded at a density of 6,000 cells per well (100 μL) in 96-well cell plates. After an overnight incubation, GFI was introduced to the cells and incubated for 48 hours. Subsequently, 10% CCK-8 staining solution (100 μL) was added to each well and incubated for 0.5 - 3 hours at 37°C. The optical density (OD) was measured and used to calculate cell viability.

Cell apoptosis was detected using flow cytometry^28^, ^29^. Cells were treated with GFI (0, 50, 100 and 200 μg/mL) for 48 hours, and harvested. The cells were washed with cold PBS, resuspended in binding buffer, and stained with PE Annexin V and 7-AAD. The cells were analyzed by flow cytometry (FACS Canto Ⅱ, BD, USA). Percentages of the cells with PE Annexin V positive staining were considered as apoptotic, whereas 7-AAD-positive staining was considered as necrotic.

Cell cycle progression was performed as described^30^, ^31^. In brief, 4T1 cells were treated with GFI for 48 hours, at the concentrations of 0 μg/mL, 100 μg/mL and 200 μg/mL. The cells were then harvested, washed, and fixed with ice-cold 75% ethanol at 4°C for 4 hours. After being washed twice with ice-cold PBS, the cells were resuspended and stained with propidium iodide (25 μg/mL) at room temperature for 10 minutes. The cells were analyzed by flow cytometry. Cell cycle distribution was calculated using ModFit LT.

### Single-Cell processing for flow cytometry

Single-Cell suspension for flow cytometry was prepared from the tumor tissues, bone marrow, peripheral blood, and spleen. Tumors were resected at the end-point of the experiments and collected in ice-cold PBS for immune cells analysis. In brief, the tumors were cut into small pieces (around 1 mm^3^) and transferred to 50 mL tubes containing 2 mL of RPMI-1640 with 0.1% collagenase type I, 0.2% dispase type I and 0.2 mg/mL DNAse I. Tissues were incubated for 30-60 minutes in a 37°C shaking incubator, and filtered through a 70 μm nylon mesh. The cell suspension was centrifuged for 5 minutes at 350g, discarded the supernatant, and re-suspended in 1 mL RBC lysis buffer for 5 minutes at 37 °C, and washed with 10 mL FACS buffer (PBS containing 2% FBS). The cell suspension was subjected to centrifugation at 350 xg for 5 minutes, and the supernatant was discarded. The cell pellet was resuspended in 5 mL FACS buffer, and the number of viable cells in each sample was determined using trypan blue staining. The cells with >90% cell viability were used for further analysis. Single-cell suspensions were obtained for flow cytometry analysis.

Whole bone marrow cells were flushed with cold PBS from femurs of control tumor-free mice, control tumor mice and GFI treated mice, and single-cell suspensions were obtained by filtering through a 70-μm nylon mesh.

Spleen tissues were dissociated using a rubber pestle in 1.5 mL tube with 1 x PBS, and single-cell suspension was obtained by filtering through a 70 μm nylon mesh.

Blood was drawn and collected in 0.5 M EDTA-coated tubes. The erythrocytes of all the samples were lysed by incubating with RBC lysis buffer at room temperature for 10 min, and washed with FACS buffer by centrifugation at 350 g for 5 minutes. Pelleted cells were resuspended with FACS buffer. Single-cell suspensions were obtained for flow cytometry analysis.

### Flow cytometry

A single-cell suspension was prepared as described in the preceding section. The cells were incubated with mouse Fc receptor blocking antibody in the FACS buffer for 30 minutes on ice, followed by staining with the fluorochrome-labelled anti-mouse antibodies for 30 min on ice in the dark, and then washed with FACS buffer twice. In this study, for cell surface protein staining, the cells were incubated with 7-AAD to assess cell viability, followed by washing with FACS buffer once. The cells were resuspended with FACS buffer. All the samples were acquired on a BD FACS Canto II. All the data were analyzed using FlowJo software.

The following antibodies were used for immune profiling:

Immune cell phenotyping panel 1: CD45- APC-eFluor 780, CD11b-APC, Ly-6G/Ly-6C(GR-1)- PE-Cyanine7, CD3e- FITC, F4/80-PE and 7-AAD.

Myeloid cell phenotyping panel 2: CD45- APC-eFluor 780, CD11b-APC, Ly6G-FITC, Ly6C- PE-Cyanine7 and 7-AAD.

Lymphoid cell phenotyping panel 3: CD45- APC-eFluor 780, CD3e- FITC, CD4-APC, CD8-PE, CD19- PE-Cyanine7 and 7-AAD.

All the information for the antibodies is listed in the supplementary Table S1.

### Immunohistochemical staining

Tumor tissue and spleen samples were fixed in 10% neutral buffered formalin and then embedded by paraffin as described 32. Serial paraffin sections were prepared and then stained with H&E. Immunohistochemical staining was performed using antibodies against CD11b, Ly6G, CD3, CD4, CD8, TIGIT, and Granzyme B. In brief, all the tissue sections were de-waxed in xylene and rehydrated in graded ethanol. For antigen retrieval, the slides were treated with a pre-heated citrate buffer in a pressure cooker. The slides were blocked in 3% hydrogen peroxide solution for 5 minutes at room temperature. To detect MDSCs, the tissue slides were incubated with rat anti-mouse CD11b and Ly6G mAb. To detect T cells, the tissue slides were incubated with rat anti-mouse CD3^+^, CD4^+^ and CD8^+^ mAb. To detect CD8^+^T cells depletion, the tissue slides were incubated with rat anti-mouse CD8^+^ and TIGIT mAb. Subsequently, the secondary antibody Labelled Polymer-HRP (ab6721, Abcam) was used to detect primary antibodies. The DAB kit (Servicebio, China) was used to detect the secondary antibody. All the sections were counterstained with Mayer Hematoxylin, dehydrated using graded ethanol, vitrificated by dimethylbenzene and mounted with mounting medium. Washing was performed with PBS between all these steps. Images of the sections were observed and obtained using a microscope.

All the information related to the antibodies is listed in the supplementary Table S1.

### RNA extraction, RNA sequencing, and RT-PCR

Total RNA was extracted using TRIzol method, and its concentration was tested by NanoPhotometer N120 (IMPLEN, Germany). Real-time PCR was performed as described previously 33, 34. The primers are listed in the supplementary Table S3. RNA sequencing was performed by BMK (www.biomarker.com.cn). Sequencing libraries were generated using NEBNext UltraTM RNA Library Prep Kit for Illumina (NEB, USA) following the manufacturer's recommendations, and index codes were added to attribute sequences to each sample, sequenced using a NovaSeq 6000 (Illumina) according to the manufacturer's instructions.

### Statistical analysis

Error bars represent the standard deviation (SD) when not otherwise specified (n = 10 or 6). Tumor-inhibition rate values are presented as means ± standard error of the mean (SEM). Significance values were determined using t-tests, and significance levels were set as follows: *p < 0.05, **p < 0.01, #p < 0.05, and ##p < 0.01. In this context, ' * ' denotes comparisons between the non-tumor normal group and the Tumor + 0.9% saline group, while ' # ' signifies comparisons between the Tumor + 0.9% saline control group and the GFI treatment group.

## Results

### *Grifola frondosa* polysaccharide (GFI) inhibits tumor growth *in vivo*

**Figure 1:**
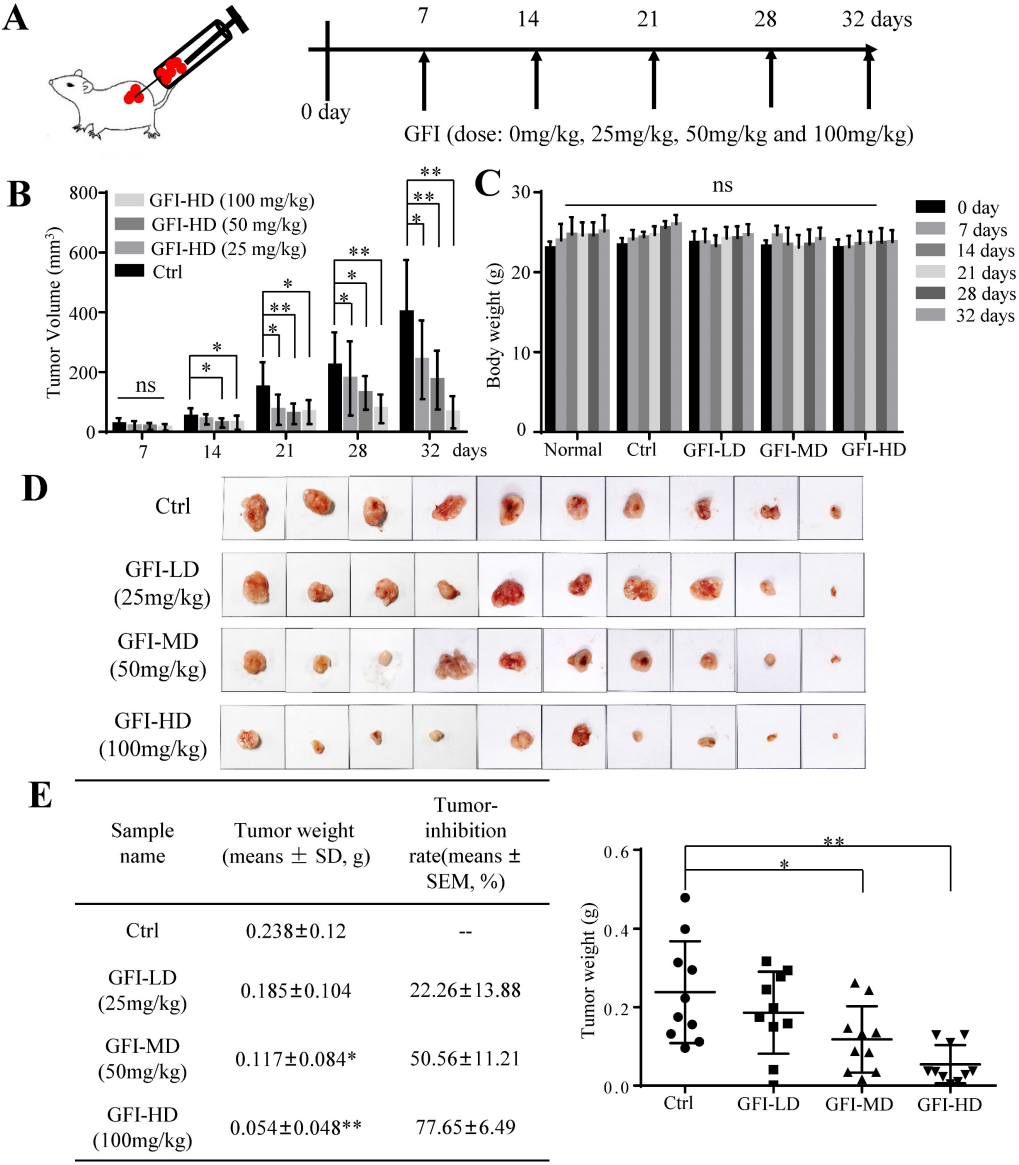
***Grifola frondosa* polysaccharide suppresses breast cancer growth in the murine models.** (A) Experimental procedures. GFI antitumor assay was performed using 4T1 cells xenografted mice tumors (0.5x10^5^ cells/each mouse). At one week after tumor inoculation, the mice were given intraperitoneal injections of GFI (25, 50, 100 mg/kg) once every other day up to 32 days, with 100 μL. Control group were given intraperitoneal injections of 0.9% saline. (B) The tumor volumes of the mice in each group of the 4T1 model were measured and calculated at the indicated time points (n = 10). (C) The body weight of mice in each group of the 4T1 model were weighed and calculated at the indicated time points (n = 10). (D) Representative photos showing the effect of GFI on tumor growth inhibition in the 4T1 murine model (n = 10). Shown on the left is the tumor weight (means ± SD) and tumor-inhibition rate (means ± SEM) in each group of the 4T1 model at the end of assay (n = 10); (E) Shown on the right is the tumor weight of each mouse in each group (n = 10). *p<0.05; **p<0.01. Error bars (no specifically indicated), SD.

To investigate the effect of GFI on tumor growth, we first established 4T1 murine breast cancer model. The mice were given intraperitoneal injections of GFI (25, 50, 100 mg/kg) once every other day starting from day 7 after tumor inoculation, and the process was repeated for 32 days (Fig. 1A). Tumor growth curve showed that GFI treatment can significantly inhibit the growth of 4T1 cells in mice in a dose- and time-dependent manner from 21 days post tumor inoculation compared with the control group (Fig. 1B; p<0.05). During treatment, the body weight of the mice showed no significant difference among all the groups (Fig. 1C), indicating no toxicity of GFI treatment. By the end of the experiment, the tumor weight in the three groups with GFI treatment was lower than the control group, and the average inhibitory rate was 22.26±13.88%, 50.56±11.21% (p<0.05) and 77.65±6.49% (p<0.01), respectively (Fig. 1D and 1E). Therefore, the significant tumor suppressive response to GFI was at the dose between 50 and 100 mg/kg, which is a feasible dose for potential drug. These data suggested that the pharmacological activation of GFI could effectively suppress tumor growth in mice, thus exhibiting a dose-effect relationship.

### GFI has no effect on tumor cell viability *in vitro*

**Figure 2:**
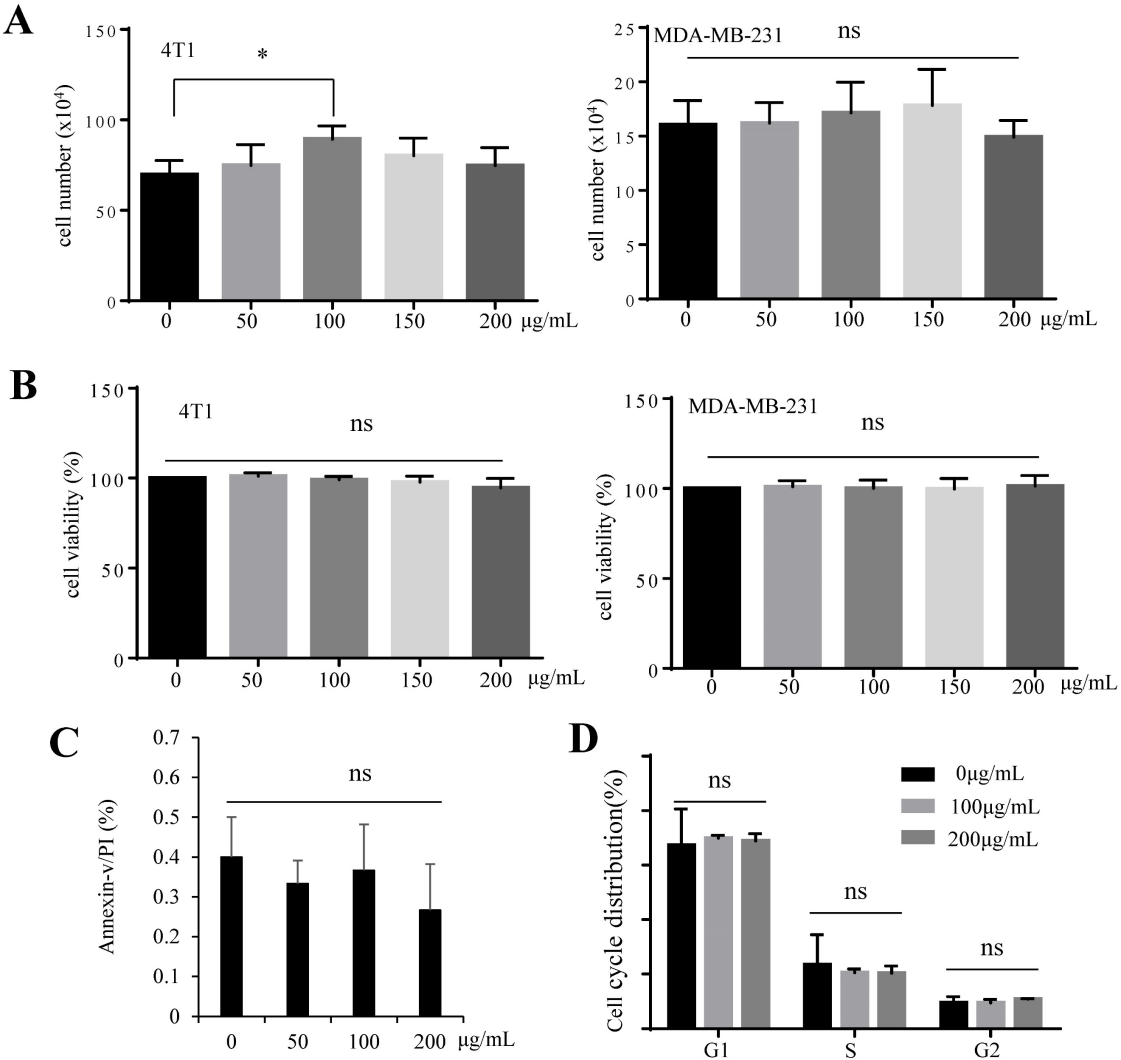
***Grifola frondosa* polysaccharide does not induce apoptosis of 4T1 cells *in vitro*.** (A) GFI was added to the cultures of 4T1 and MDA-MB-231 cells at the concentrations of 0, 50, 100, 150, 200 μg/mL, followed by 48 hours incubation. Cell proliferation was analyzed by trypan blue staining and cell counting. Each experiment was repeated three times. (B) 4T1 cells were treated with GFI for 48 hours. Next, 10% CCK-8 staining (100 μL) was added to each well and incubated for 0.5 - 3 hours, at 37°C. The optical density (OD) value was measured and calculated. Each experiment was repeated three times. (C) 4T1 cells were treated with GFI (0, 50, 100, 200 μg/mL) for 48 hours, followed by flow cytometry using propidium iodide (PI)/Annexin V staining. Each experiment was repeated three times. (D) 4T1 cells were treated with GFI (0, 100, 200 μg/mL) for 48 hours. The cell cycle of 4T1 cells was detected with PI staining by flow cytometry. *p<0.05; **p<0.01. Error bars, SD.

We investigated whether the tumor suppressive effect of GFI is mediated by inducing tumor cell apoptosis. Cell proliferation analysis by trypan blue staining showed that GFI did not induce 4T1 and MDA-MB-231 cells death (Fig. 2A). Treatment of cells with GFI did not reduce 4T1 and MDA-MB-231 cell viability by CCK-8 assay (Fig. 2B). Cell apoptosis in both 4T1 cells treated with GFI and the control did not reveal any change as studied by flow cytometry of Annexin V/propidium staining analysis, indicating the GFI treatment did not induce apoptotic cell death (Fig. 2C, Fig. S3A). Furthermore, GFI showed no effect on cell cycle distribution by flow cytometry (Fig. 2D, Fig. S3B). Thus, we postulate that GFI might not directly induce programmed tumor cell death to exert its tumor inhibitory effects.

### GFI treatment inhibits MDSCs infiltration into the tumor site

**Figure 3:**
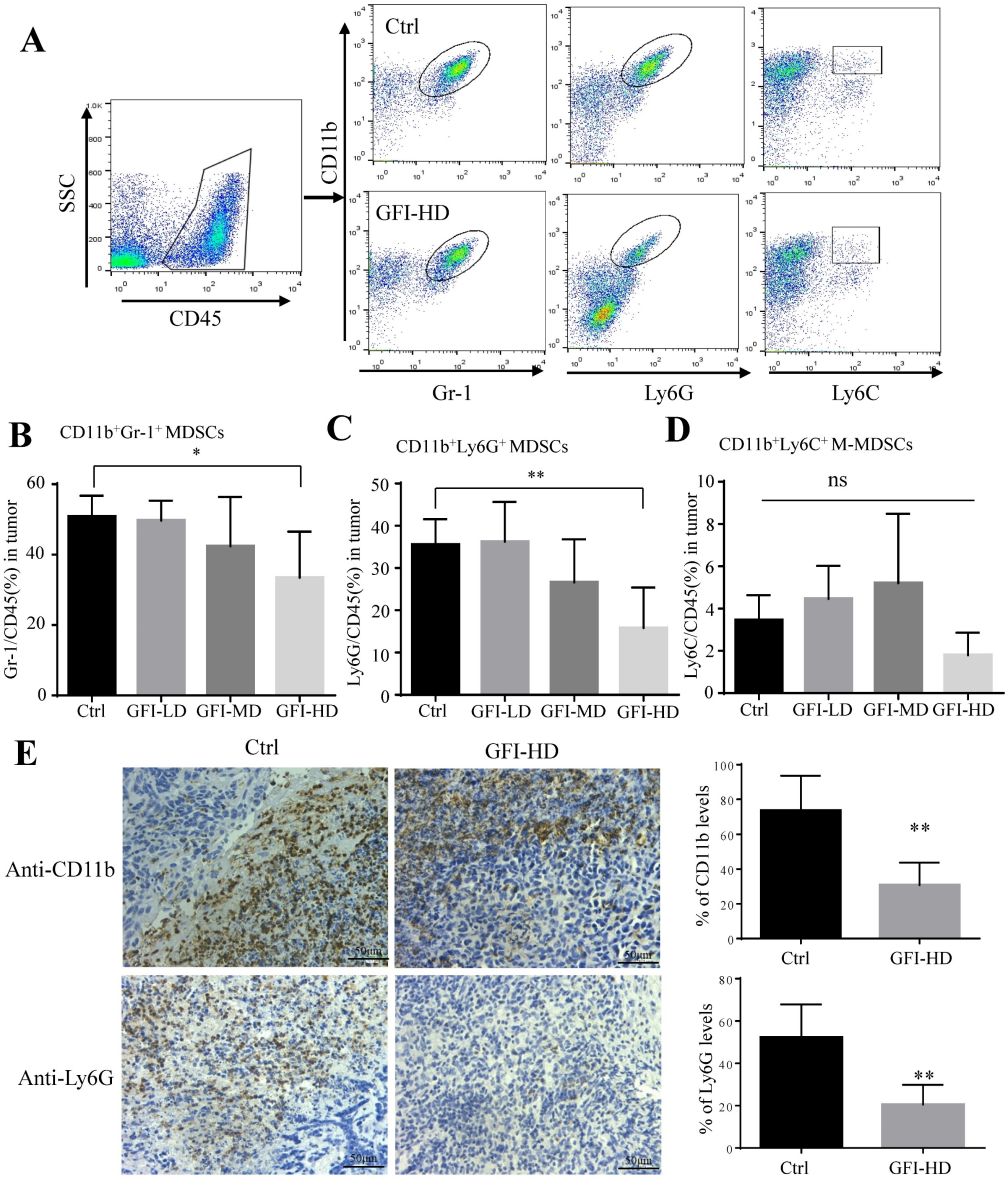
***Grifola frondosa* polysaccharide suppresses the infiltration of MDSCs in the tumors.** (A) Representative FACS plots illustrate the gating to assess Gr-1^+^, Ly6G^+^ and Ly6C^+^ cells in the tumor from the control or GFI-treated 4T1 murine model. (B-D) After mice were sacrificed, single-cell suspensions were obtained from the tumor of the control or GFI-treated 4T1 mice. Single-cell suspensions were stained with MDSCs cell surface markers (CD45, CD11b, Gr-1(Ly6G/Ly6C), Ly6G and Ly6C antibodies). MDSCs were detected by flow cytometry. Summary of the % of MDSCs (CD45^+^Gr-1^+^), PMN-MDSCs (CD45^+^CD11b^+^ Ly6G^+^) and M-MDSCs (CD45^+^CD11b^+^Ly6C^+^) in tumor of the control or GFI-treated 4T1 mice (n = 5). (E) Left, Immunohistochemistry staining showed the protein expression level of CD11b and Ly6G in the tumors of the control or GFI-treated 4T1 mice. Right, Relative protein expression level of the % of CD11b and Ly6G was calculated by image-pro plus in tumor of the control or GFI-treated 4T1 mice. *p<0.05; *p<0.01. Error bars, SD.

Next, we examined the immune cell profile alterations in the 4T1 mice after GFI treatment to identify whether the antitumor immune response in the tumor microenvironment involved in the tumor suppression effects of GFI. First, we analyzed the alterations of the immunosuppressive cells CD11b^+^Gr-1^+^ MDSCs, CD11b^+^Ly6G^+^ PMN-MDSCs, CD11b^+^ Ly6C^+^ M-MDSCs and CD11b^+^F4/80^+^ TAMs using flow cytometry and immunohistochemical analysis (Fig. 3A, Fig. S4A). Interestingly, it was found that CD11b^+^ myeloid cells were significantly reduced in 4T1 tumor-bearing mice with GFI treatment compared to the untreated ones (Fig. 3, Fig. S4), which might be due to the decrease of CD11b^+^Gr-1^+^ MDSCs percentage with high dose GFI treatment (Fig. 3B), but not the CD11b^+^F4/80^+^ TAMs (Fig. S4C). Gr-1 antibody can recognize two antigens Ly6G and Ly6C, which expressed on two distinct subsets of Gr-1^+^ myeloid cells, polymorphonuclear CD11b^+^Ly6G^+^ (PMN-MDSCs) and monocytic CD11b^+^Ly6C^+^ (M-MDSCs), both subtypes supporting tumor growth and suppressing antitumor immunity^35^. We found that CD11b^+^Ly6G^+^PMN-MDSCs were the major CD11b^+^Gr-1^+^ cells subset in the tumor tissue which were significantly decreased in the tumor after GFI treatment in a dose-dependent manner (Fig. 3C, 3D). Immunohistochemical analysis further proved that GFI reduced the expression of Ly6G in the tumor of 4T1 murine models compared with the controls (Fig. 3E). These results demonstrated that GFI inhibited MDSCs infiltration and enrichment in the breast tumors.

### GFI treatment decreased MDSCs in the peripheral blood

**Figure 4:**
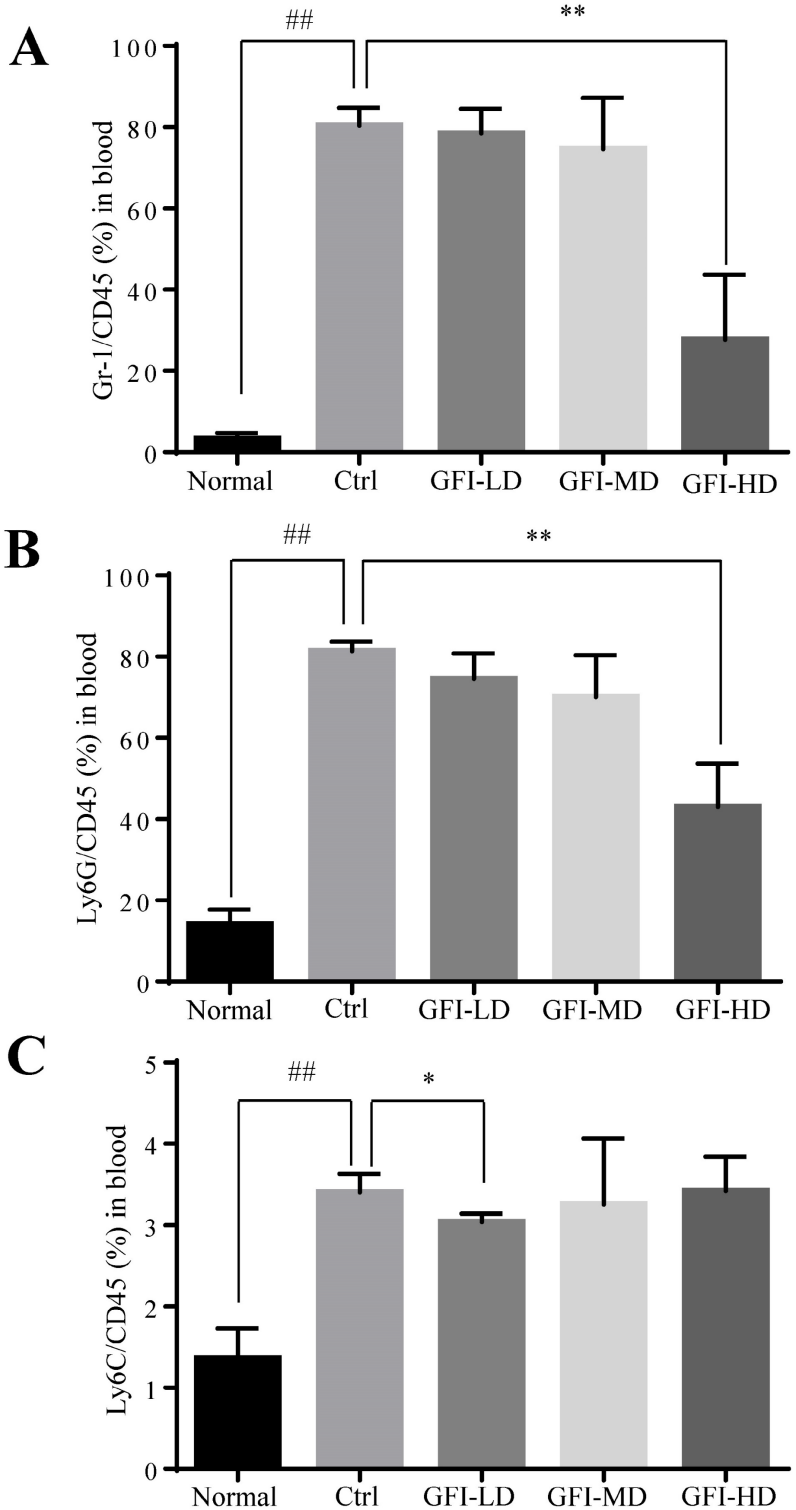
***Grifola frondosa* polysaccharide suppresses the enrichment of MDSCs in the peripheral blood.** (A) After the mice were sacrificed, MDSCs were analyzed with cell surface markers (CD45^+^CD11b^+^ Gr-1^+^) in peripheral blood from the control or GFI-treated 4T1 murine model using flow cytometry (n = 4). GFI treatment decreased Gr-1^+^MDSCs enrichment in the peripheral blood as observed by using flow cytometry analysis. (B-C) Flow cytometry analyzed the density of PMN-MDSCs (B) and M-MDSCs (C) in the peripheral blood from the control or GFI-treated 4T1 murine model, staining with CD45, CD11b, Ly6G and Ly6C antibodies (n = 4). *p<0.05; *p<0.01; #p<0.05; ##p<0.01; Error bars, SD.

In patients with cancer, MDSCs were reported to be increased in the circulation and correlated with tumor burden^36^, ^37^. Here, we used the 4T1 tumor-bearing mouse model that has previously been demonstrated to show MDSCs expansion in the circulation, spleen and the tumor tissues 38, 39. We then investigated the effect of GFI on the MDSCs in the peripheral blood of mice with tumors using flow cytometry analysis. The results showed that high dose of GFI treatment reduced the percentage of Gr-1^+^ MDSCs in the peripheral blood of mice with tumors compared with the untreated control group (Fig. 4A). PMN-MDSCs were the dominant subset population in the peripheral blood (Fig. 4B). GFI treatment decreased the percentage of PMN-MDSCs in a dose dependent manner (Fig. 4B, GFI-HD 100 mg/kg p<0.001). In addition, GFI treatment also reduced M-MDSCs in the blood (Fig. 4C).

### GFI treatment suppressed splenomegaly and MDSCs accumulation in the spleen

**Figure 5:**
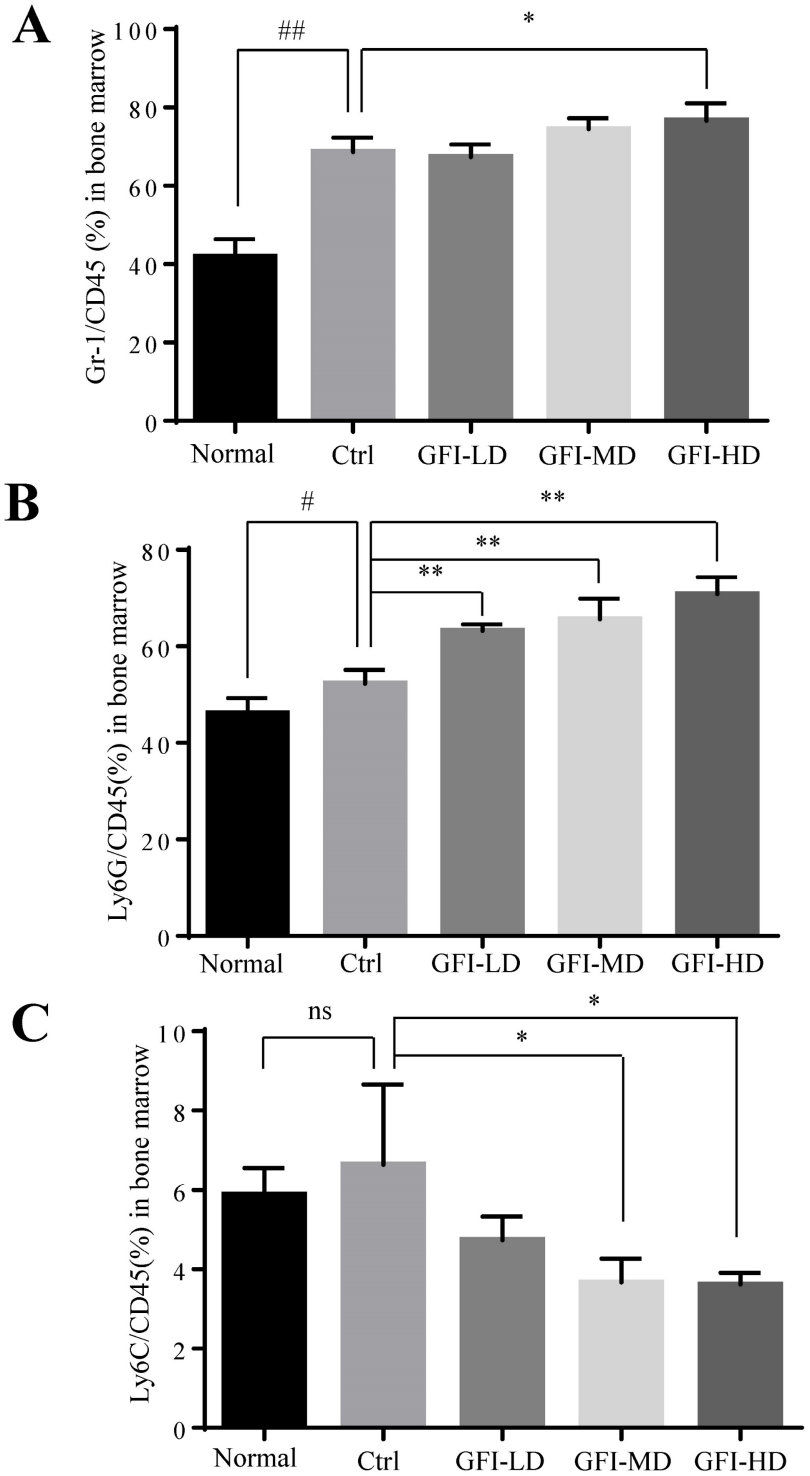
***Grifola frondosa* polysaccharide did not suppress the expansion of MDSCs in the bone marrow.** (A) After the mice were sacrificed, MDSCs were analyzed with the cell surface markers (CD45^+^CD11b^+^ Gr-1^+^) in the bone marrow from the normal, control or GFI-treated 4T1 murine model using flow cytometry (n = 4). GFI treatment did not suppress Gr-1^+^ MDSCs expansion in the bone marrow as seen through flow cytometry analysis. (B-C) Flow cytometry analyzed the density of PMN-MDSCs (B, CD45^+^CD11b^+^Ly6G^+^) and M-MDSCs (C, CD45^+^CD11b^+^Ly6C^+^) in the bone marrow from the normal, control or GFI-treated 4T1 murine model, staining with CD45, CD11b, Ly6G and Ly6C antibodies (n = 4). *p<0.05; *p<0.01; #p<0.05; ##p<0.01; Error bars, SD.

It has been acknowledged that MDSCs are also produced in the peripheral organ spleen while they proliferate in the bone marrow^24^, ^40^. Here, we investigated the effect of GFI on MDSCs in bone marrow and spleen of the mice with tumors using flow cytometry analysis. The FACS analysis showed that the immune suppressive cells MDSCs, PMN-MDSCs and M-MDSCs were all increased in the bone marrow of tumor-bearing mice compared with the normal group (Fig. 5). GFI treatment did not suppress the expansion of MDSCs, PMN-MDSCs in the bone marrow of 4T1 mice compared with the control group (Fig. 5A, 5B), but decreased the percentage of Ly6C+ M-MDSCs (Fig. 5C).

**Figure 6:**
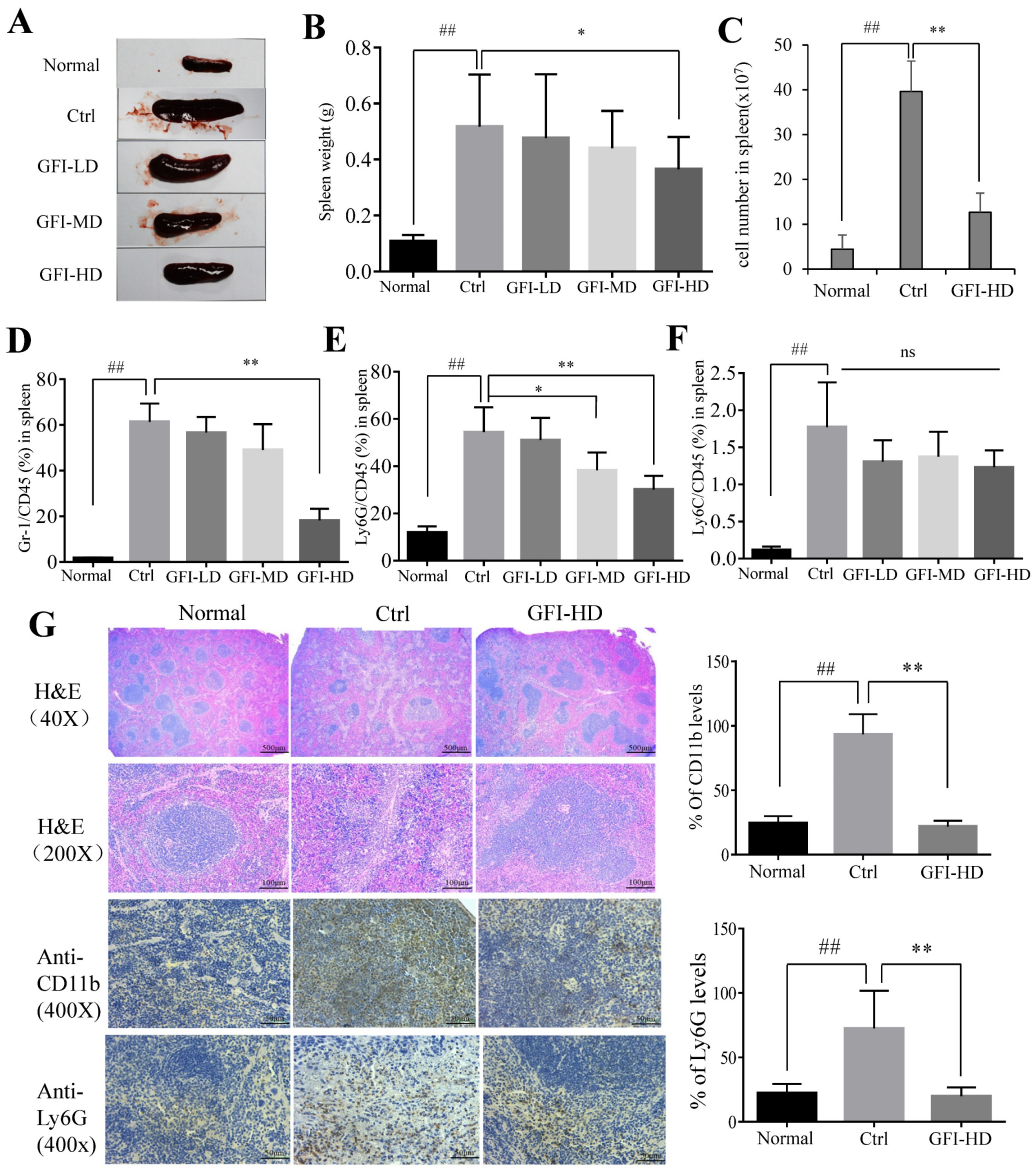
***Grifola frondosa* polysaccharide suppresses the expansion of MDSCs in the spleen.** Representative images of spleens. After mice were sacrificed, the spleen weights of the mice in each group were measured and calculated at the end of the assay (n = 10). After mice were sacrificed, a single cell suspension was obtained from the spleens of normal, control and GFI-treated mice (n = 3). Total cell number in spleens were counted and calculated. After the mice were sacrificed, MDSCs were analyzed with the cell surface markers (CD45^+^CD11b^+^Gr-1^+^) in the spleens from the normal, control or GFI-treated 4T1 murine model using flow cytometry (n = 4). GFI treatment decreased Gr-1^+^ MDSCs enrichment in the spleen as observed through flow cytometry analysis. GFI treatment decreased Ly6G^+^ PMN-MDSCs enrichment in the spleen using flow cytometry analysis (n = 4). GFI treatment decreased Ly6C^+^ M-MDSCs enrichment in the spleen using flow cytometry analysis (n = 4). Left, H&E, CD11b and Ly6G staining of the spleen from the normal, control or GFI-treated 4T1 murine model. Right, relative protein expression level of the % of CD11b (upper) and Ly6G (lower) was calculated by Image-pro plus. *p<0.05; *p<0.01; #p<0.05; ##p<0.01; Error bars, SD.

In agreement with the previous reports on 4T1 murine models^39^, ^41^, the spleen became enlarged in untreated tumor-bearing mice, resulting in 3.79-fold increase in weight (0.517 g) compared to the normal mice (0.107 g), whereas GFI treatment effectively suppressed spleen enlargement in 4T1 tumor-bearing mice (GFI-HD: 0.365 g) (Fig. 6A, 6B). It has been previously reported that splenomegaly in both cancer patients and tumor-bearing mice was caused by the expansion of CD11b+Gr-1+ myeloid immuno-suppressor cells and was positively correlated with tumor size^1^, ^2^. The number of splenocytes in the spleens of 4T1 mice increased 7.95-fold more than the normal mice, while the number of splenocytes were decreased after GFI treatment (Fig. 6C). FACS data showed that CD11b+Gr-1+MDSCs significantly increased in the spleen of untreated tumor-bearing mice compared with the normal mice (up to 61.3 ± 8.02% vs 1.81 ± 0.19%), while the content of MDSCs in the spleen of tumor bearing mice after GFI treatment decreased to 56.6 ± 6.74% (GFI-LD), 49.1 ± 11.2% (GFI-MD) and 18.2 ± 5.14%, respectively (Fig. 6D).

Consequently, it was found that CD11b+ Ly6G+ PMN-MDSCs were the major type among MDSCs, and the percentage of Ly6G/CD45 decreased to 51.0±9.34%, 38.2±7.55% and 30.1±5.74% of MDSCs after GFI treatment in the spleen compared with the control (Ly6G/CD45: 54.4±10.51%) (Fig. 6E, 6F). In accordance, H&E analyses of spleen tissues showed indistinct boundaries between the red and white pulps in the spleens of the model mice, which indicated that the structure of spleen tissues in the mice receiving GFI was restored similar to that in the control mice (Fig. 6G). Moreover, immuno-histochemical analysis proved that Ly6G+ MDSCs were decreased in the spleen of 4T1 murine models with GFI treatment compared with the control (Fig. 6G). Therefore, we concluded that GFI treatment could inhibit the expansion and accumulation of MDSCs in the spleen of the tumor-bearing mice.

### GFI treatment activates antitumor immune response in mice

**Figure 7:**
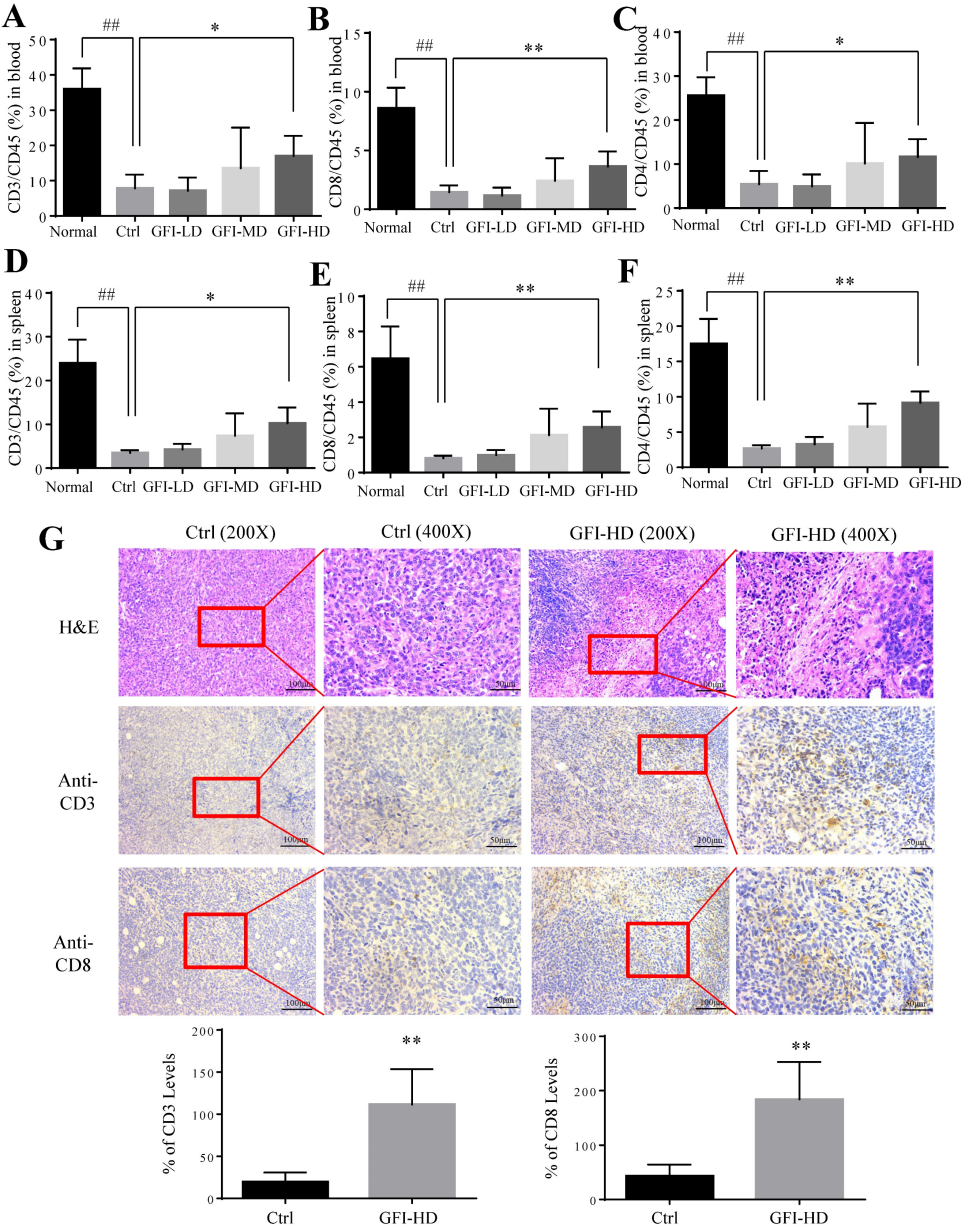
***Grifola frondosa* polysaccharide increased T cells in the peripheral blood, spleen and tumors.** After mice were sacrificed, a single cell suspension was obtained from the blood and spleens of normal, control and GFI-treated mice. Single cell suspensions were stained with T lymphocyte cell surface markers (CD45, CD3, CD8 and CD4 antibodies). Stained cells were detected by flow cytometry. (A), (B) and (C) GFI increased the percentage of CD3^+^ (A), CD8^+^ (B), and CD4^+^ (C) T cells in the peripheral blood from the normal, control or GFI-treated 4T1 murine model (n = 4). (D), (E) and (F) GFI increased the percentage of CD3^+^ (D), CD8^+^ (E), and CD4^+^ (F) T cells in the peripheral blood from the normal, control or GFI-treated 4T1 murine model (n = 4). (G) Upper, H&E, CD3 and CD8 staining of the tumors from the control and GFI-treated 4T1 murine model. Lower, Relative protein expression level of the % of CD3 (left) and CD8 (right) was calculated by Image-pro plus in tumor of the control or GFI-treated 4T1 mice. *p<0.05; *p<0.01; #p<0.05; ##p<0.01; Error bars, SD.

It has been reported that MDSCs potently inhibit T-cell-mediated antitumor immunity in the cancer patients and the tumor murine models^2^, ^35^. Therefore, we investigated whether GFI may affect the antitumoral T cells responses in the peripheral blood, spleen and tumor site. Interestingly, the number and percentages of CD3^+^, CD4^+^ and CD8^+^ T lymphocytes were significantly increased in the spleen and blood of GFI-treated mice (Fig. 7A - 7F). Consistently, GFI treatment increased the populations of CD3^+^ and CD8^+^ T lymphocytes at the tumor site in the tumor-bearing mice (Fig. 7G).

**Figure 8:**
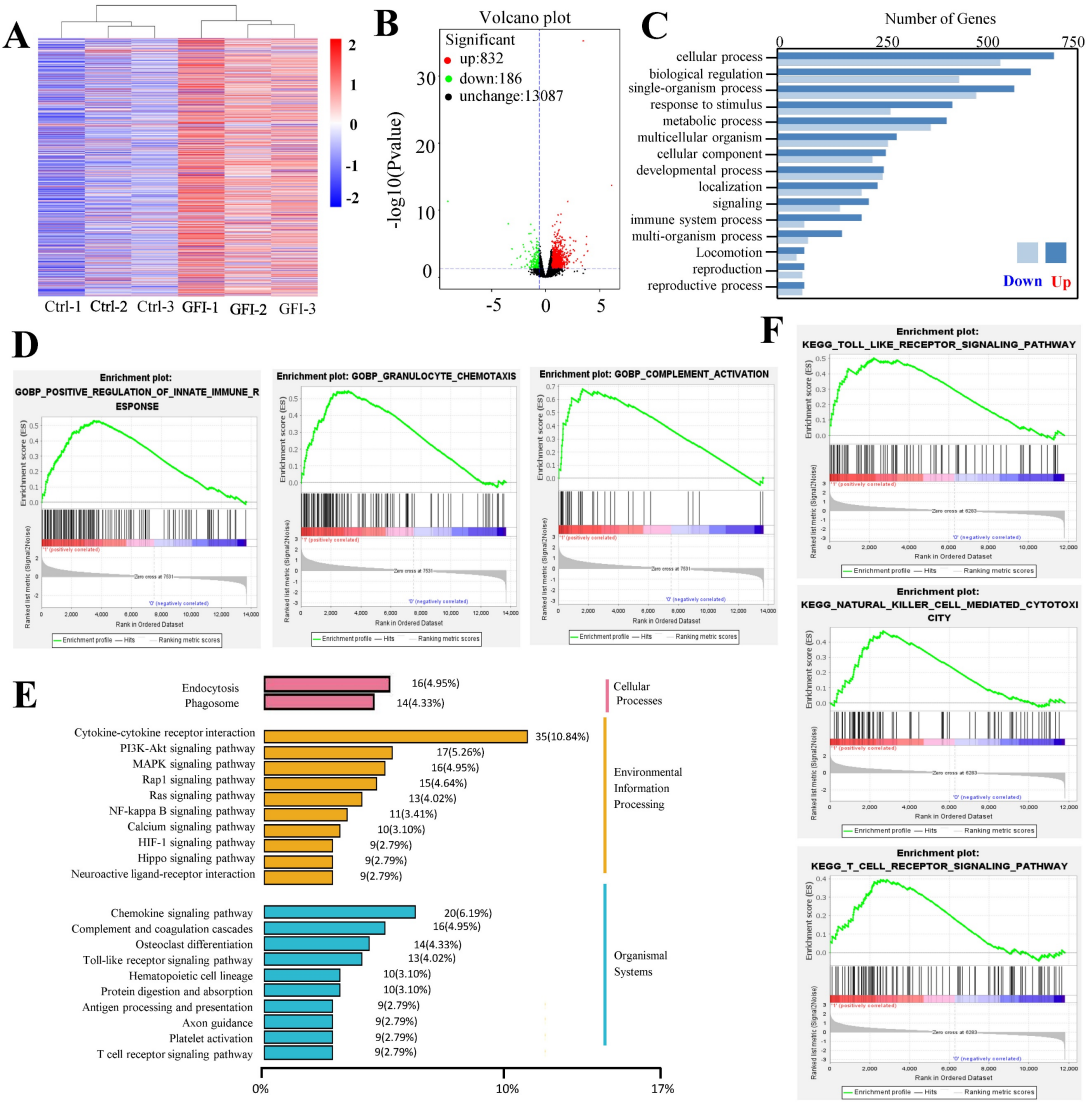
***Grifola frondosa* polysaccharide activated immune response.** A comparison of mRNA level in the tumor of GFI group and control group by using RNA sequencing (n = 3). (A) Heatmap showing the differentially expressed mRNAs in tumor treated with control and GFI. (B) Volcano plots of differentially expressed genes in tumor from GFI-treated mice compared to control mice (-log10 (P value) ≤0.05 and fold change (FC≥1.5)). (C) Gene Ontology (GO) Categories biological process results showed the genes of immune system process were enriched in tumor of GFI-treated mice versus control mice. (D) GSEA for all genes results related to gene sets in innate immune response activation, granulocyte chemotaxis and complement activation in tumor of GFI-treated mice versus control mice. (E) KEGG class results showed the genes of phagosome, toll like receptor, T cell receptor and NK cell mediated cytotoxicity were enriched in tumor of GFI-treated mice versus control mice. (F) GSEA results showed that the genes of the antitumor immune response activation pathway were enriched in tumor of GFI-treated mice versus control mice.

Because analysis of intratumoral immune cell populations displayed quantitative changes by GFI treatment, we further analyzed the potential alterations of gene expression and thus performed RNA sequencing in tumors harvested from mice that were treated with GFI or 0.9% saline. The sequencing analysis showed that GFI treatment could trigger the alterations of genes at the mRNA level, 1018 differentially expressed genes (p< 0.05) with 832 genes higher and 186 genes lower in the tumor samples of 4T1-mice treatment compared to control (Fig. 8A, 8B). Subsequent of these gene were enriched on immune system (GO term biological process) (Fig. 8C). In addition, we performed gene set enrichment analysis (GSEA) to study the function of all gene as described 42, 43. Immune response pathways such as innate immune response activation, complement activation and granulocyte chemotaxis were significantly enriched in GFI group (Fig. 8D). The data analysis also found gene expression linked to the phagocytic function in tumor from GFI-treated mice was enhanced (Fig. 8E). The phagocytic function of innate immunity is related to its antitumor activity^44^. Moreover, gene set enrichment analysis also revealed that the pathway involved in immune response activation were positively enriched in toll like receptor, T cell receptor and NK cell mediated cytotoxicity (Fig. 8F), indicating that GFI enhances antitumor immune response in the 4T1-bearing mice.

**Figure 9:**
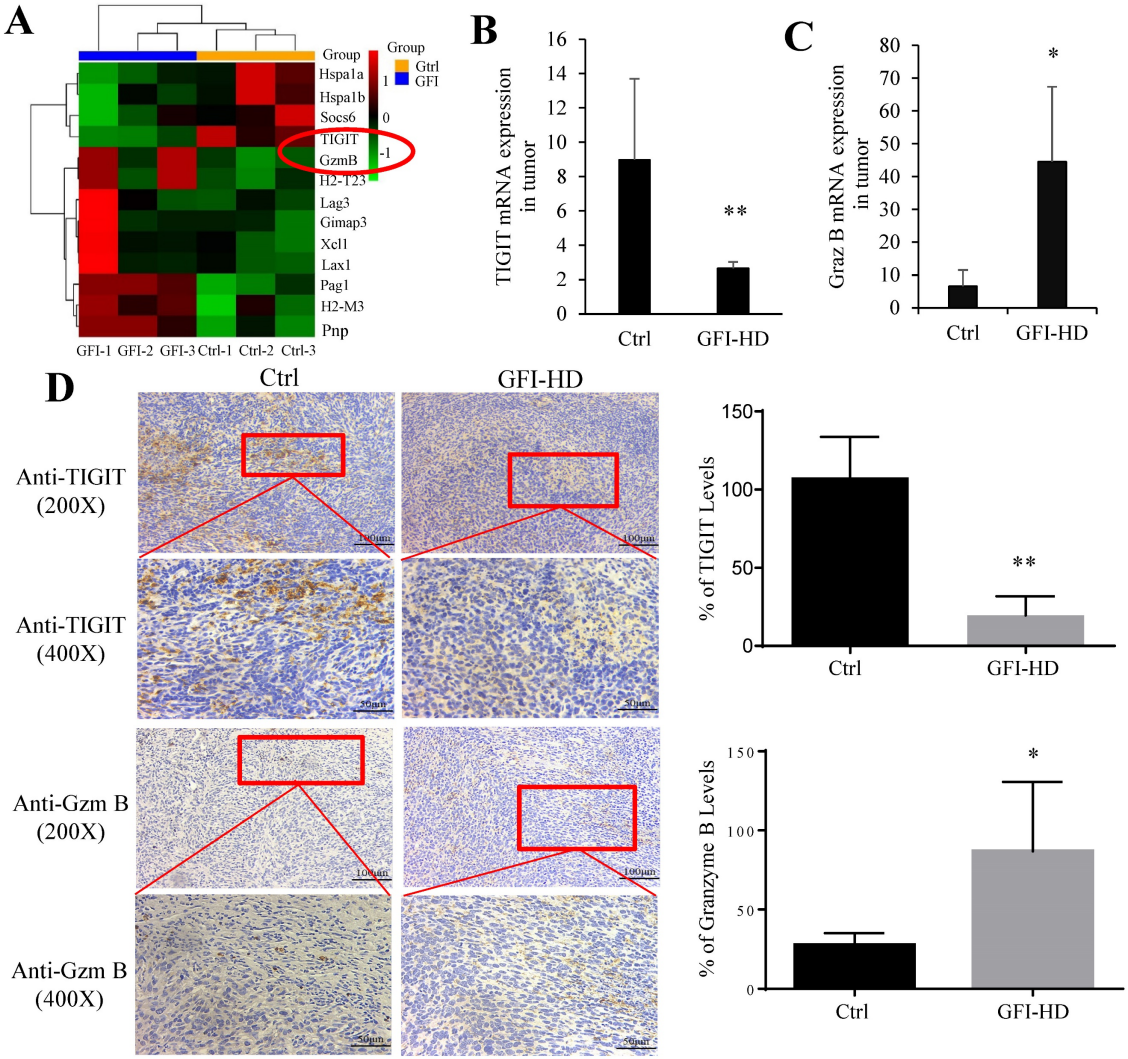
***Grifola frondosa* polysaccharide activated CD8^+^ T cells in the tumors.** (A) Heatmap of the genes obtained from T cell-mediated cytotoxicity and negative regulation of T cell activation in RNA-seq data. (B-C) qPCR assays to measure the mRNA levels of TIGIT (B) and granzyme B (C) in control-treated and GFI-treated mice. (D) Left, Immunohistochemistry staining showed the protein expression level of TIGIT and granzyme B in the tumors of the control or GFI-treated 4T1 mice. Right, Relative protein expression level of the % of TIGIT (upper) and granzyme B (lower) was calculated by Image-pro plus in tumor of the control or GFI-treated 4T1 mice. *p<0.05; *p<0.01; #p<0.05; ##p<0.01; Error bars, SD.

In the tumor microenvironment, MDSCs can hinder T cell effector functions. To gain deeper insights into the molecular mechanisms responsible for T cell activation by GFI, we analyzed RNA-seq data to examine the expression of genes related to T cell-mediated cytotoxicity and the negative regulation of T cell activation. Our findings revealed a significant down-regulation of TIGIT (p=0.0004) and an up-regulation of granzyme B (p=0.0002) in response to GFI treatment (Fig. 9A). TIGIT is known to contribute to the 'exhaustion' state of infiltrating T cells by binding to CD155 on MDSCs^45^. We further validated TIGIT were significantly decreased in GFI-treated 4T1 tumor-bearing mice using RT-PCR and immunohistochemical analysis (Fig. 9B, 9D). Granzyme B plays a vital role in enabling CD8^+^ T cells to eliminate cancer cells within the tumor microenvironment. Nevertheless, we detected that the mRNA and protein expression of granzyme B were elevated in GFI-treated 4T1 tumor-bearing mice (Fig. 9C, 9D). These results demonstrated that GFI treatment can restore and activate antitumor immune responses in the tumor microenvironment by enhancing activation of innate immune response, and infiltration and activation of CD8^+^T cell.

## Discussion

The recognition of the importance of tumor-induced immune suppression in cancer has led to a shift regarding approaches for cancer immunotherapy 46. MDSCs are a major component of the tumor immune cell infiltrate, and these cells are proved to weaken the efficiency of antitumor immune responses of CD4^+^ T, CD8^+^ T cells 9, 35, 47. Therefore, it has become increasingly clear that a strategy eliminating MDSCs for successful cancer therapy could act alone, or synergistically with other anticancer therapies. In this study, we have shown that GFI treatment resulted in a significant reduction in the percentage of MDSCs in the spleen, blood, and tumor tissues of mice. This reduction was found to be negatively correlated with the percentages of CD4^+^ T and CD8^+^ T cells. Additionally, it was negatively correlated with the growth of 4T1 cells in the mice. Thus, in the context of 4T1 mice, GFI, a polysaccharide-rich extract from *G. frondosa*, effectively depleted MDSCs, leading to the activation of the host's antitumor immune response.

MDSCs can be categorized into two subtypes: PMN-MDSCs and M-MDSCs, both of which play roles in suppressing antitumor immunity and promoting tumor growth. Previous research has shown that the expansion of PMN-MDSCs is associated with tumor stage and splenomegaly in breast cancer patients and mice^7^, ^15^, ^48^. In our study, GFI exhibited a concentration-dependent inhibition of splenomegaly, and this inhibition was found to have a negative correlation with the number and proportion of MDSCs. Furthermore, it was observed that the majority of these MDSCs were CD11b^+^Ly6G^+^ PMN-MDSCs. Numerous polysaccharides or polysaccharide-protein complexes from fungi have shown to produce their antitumor effect, through mechanisms that seem to enhance innate immune and cell-mediated immune responses, especially phagocytosis by macrophages 19, 49-51. However, only in the recent years, the evidence indicated that fungi-derived polysaccharides inhibited the immunosuppressive function of MDSCs in the tumor-bearing host 52, 53. The polysaccharide from* L. edodes* promoted the differentiation of CD11b^+^Ly6C^+^ M-MDSCs to inhibit tumor growth in mice 54, 55. We also observed that lentinan significantly reduced the presence of M-MDSCs in the blood and spleen of tumor-bearing mice. However, it's noteworthy that lentinan did not effectively inhibit splenomegaly (Figure S5 and S6). Lentinan is a β-D-glucan with β-1,3-, and β-1,6-glucosidic linkages and the triple-helix conformation^56^. In contrast, polysaccharides from *G. frondosa* encompass β-D-glucan, α-D-glucan and heteropolymer^57^. Consequently, the differing structural compositions of polysaccharides from *G. frondosa* and* L. edodes* may underlie their varying mechanisms of action. This may represent a novel mechanism how polysaccharides from *G. frondosa* inhibit breast cancer growth by suppressing immune suppressor cells MDSCs, mainly PMN-MDSC.

MDSCs inhibit antitumor immunity and promote tumor progression via multiple mechanisms^2^. One of the major mechanisms is that MDSCs suppress cytotoxic T cells immune responses to facilitate tumor immune escape^47^. Here, we found that GFI inhibited tumor growth in mice in a dose-dependent manner, but did not affect 4T1 cells proliferation *in vitro*. Therefore, we hypothesized that GFI may restore and activate tumor-infiltrating T lymphocytes by eliminating MDSCs. We proved that GFI increased the population of CD3^+^ and CD8^+^ T lymphocytes in the periphery and the tumor tissues of 4T1 tumor-bearing mice. TIGIT is an inhibitory regulator highly expressed in human and murine tumor infiltrating T cells^58^. Recent studies have shown that MDSCs negatively regulate T cell functions targeting TIGIT/CD155 pathway^59^. We also found that GFI reduced the mRNA and protein expression of TIGIT in the tumor tissues. Meanwhile, the levels of Granzyme B were consistently higher in CD8^+^ T cells of the GFI treated mice. Taken together, GFI eliminated MDSCs, and restored and activated CD8^+^ T cells immune response to inhibit tumor growth in the tumor microenvironment.

It is well-known that MDSCs descend from the immature myeloid cells (IMCs), which are produced in the bone marrow in the tumor-bearing host^24^. In addition to originating from bone marrow, CD11b^+^Gr-1^+^ MDSCs that accumulated in the spleen of tumor-bearing mice have also been reported to be mainly produced from the hematopoietic stem and progenitor cells of the splenic red pulp, and showed a distinct immunosuppressive phenotype^60^-^62^. GFI administration in the tumor-bearing mice, in the present study, significantly reduced the percentage and number of splenic MDSCs and increased the percentage of CD8^+^T cells in the spleen, but did not reduce the percentage of CD11b^+^Gr-1^+^ MDSCs in the bone marrow. Therefore, we concluded that GFI promoted the occupancy of spleen by the CD8^+^ T cells via actively inhibiting the replenishment and accumulation by Ly6G^+^ PMN-MDSCs, and GFI treatment reduced the tumor-infiltrating MDSCs via suppressing the expansion of splenic MDSCs. Future studies will focus on understanding how GFI inhibits MDSCs expansion in the spleen and their migration to the tumor site.

Although, polysaccharides from* G. frondosa*, as immune agonists, have been used against cancer in preclinical studies and in selected clinical patients, the underlying mechanisms have not yet been completely understood. In the future studies, we will extract and purify the components of GFI to obtain the MDSCs inhibitor and further investigate the structure and functional mechanisms to explore its potential to be employed in antitumor immune therapy. Taken together, we demonstrated a novel mechanism of GFI that inhibited breast cancer growth by suppressing immune suppressor cells to restore and reactivate antitumor immune response. We found that GFI reduced MDSCs infiltration in the tumor tissue, and consequently led to an increase in the percentage and activation of CD8^+^ T cells. Furthermore, we revealed that GFI reduced the expansion of splenic MDSCs, which resulted in the decrease of MDSCs percentage in the circulation and the tumor tissue in the tumor-bearing host. Our findings lay the foundation of the application of fungal polysaccharides, as novel agents, in anticancer therapies to combine with other agents, thus achieving more effective outcomes. Purification of the components and identifying the crystal structures of GFI components warrants further investigation in the future.

## Supplementary Material

Supplementary figures.

Supplementary tables.

## Abbreviations

GFI: polysaccharide isolated from *Grifola frondosa*
25 mg/kg): GFI-LD, low dose
50 mg/kg): GFI-MD, medium dose
100 mg/kg): GFI-HD, high dose
DMEM: Dulbecco's modified Eagle's medium
FBS: fetal bovine serum

## References

[1] Bronte V, Brandau S, Chen S-H, Colombo MP, Frey AB, Greten TF (2016). Recommendations for myeloid-derived suppressor cell nomenclature and characterization standards. Nature Communications.

[2] Li K, Shi H, Zhang B, Ou X, Ma Q, Chen Y (2021). Myeloid-derived suppressor cells as immunosuppressive regulators and therapeutic targets in cancer. Signal Transduction and Targeted Therapy.

[3] Kajihara N, Kobayashi T, Otsuka R, Nio-Kobayashi J, Oshino T, Takahashi M (2023). Tumor-derived interleukin-34 creates an immunosuppressive and chemoresistant tumor microenvironment by modulating myeloid-derived suppressor cells in triple-negative breast cancer. Cancer Immunology Immunotherapy.

[4] Bergenfelz C, Roxå A, Mehmeti M, Leandersson K, Larsson A-M (2020). Clinical relevance of systemic monocytic-MDSCs in patients with metastatic breast cancer. Cancer Immunology, Immunotherapy.

[5] Jimenez-Cortegana C, Galluzzi L (2023). Myeloid-derived suppressor cells: Emerging players in cancer and beyond. International review of cell and molecular biology.

[6] Kanterman J, Sade-Feldman M, Biton M, Ish-Shalom E, Lasry A, Goldshtein A (2014). Adverse immunoregulatory effects of 5FU and CPT11 chemotherapy on myeloid-derived suppressor cells and colorectal cancer outcomes. Cancer research.

[7] Youn JI, Nagaraj S, Collazo M, Gabrilovich DI (2008). Subsets of Myeloid-Derived Suppressor Cells in Tumor-Bearing Mice. Journal of Immunology.

[8] Mackert JR, Qu P, Min Y, Johnson PF, Yang L, Lin PC (2017). Dual negative roles of C/EBPα in the expansion and pro-tumor functions of MDSCs. Scientific Reports.

[9] Vanhaver C, Bruggen P, Bruger AM (2021). MDSC in Mice and Men: Mechanisms of Immunosuppression in Cancer. Journal of Clinical Medicine.

[10] Rong Y, Yuan CH, Qu Z, Zhou H, Guan Q, Yang N (2016). Doxorubicin resistant cancer cells activate myeloid-derived suppressor cells by releasing PGE2. Scientific Reports.

[11] Eriksson E, Wenthe J, Irenaeus S, Loskog A, Ullenhag G (2016). Gemcitabine reduces MDSCs, tregs and TGFβ-1 while restoring the teff/treg ratio in patients with pancreatic cancer. Journal of Translational Medicine.

[12] Kodumudi KN, Woan K, Gilvary DL, Sahakian E, Wei S, Djeu JY (2010). A Novel Chemoimmunomodulating Property of Docetaxel: Suppression of Myeloid-Derived Suppressor Cells in Tumor Bearers. Clinical Cancer Research An Official Journal of the American Association for Cancer Research.

[13] Liu D, You M, Xu YJ, Li FL, Zhang DY, Li XJ (2016). Inhibition of curcumin on myeloid-derived suppressor cells is requisite for controlling lung cancer. International Immunopharmacology.

[14] Balog J, Hackler L Jr, Kovács AK, Neuperger P, Alföldi R, Nagy LI (2019). Single Cell Mass Cytometry Revealed the Immunomodulatory Effect of Cisplatin Via Downregulation of Splenic CD44+, IL-17A+ MDSCs and Promotion of Circulating IFN-γ+ Myeloid Cells in the 4T1 Metastatic Breast Cancer Model. Int J Mol Sci.

[15] Hamilton MJ, Banáth JP, Lam V, Lepard NE, Krystal G, Bennewith KL (2012). Serum inhibits the immunosuppressive function of myeloid-derived suppressor cells isolated from 4T1 tumor-bearing mice. Cancer immunology, immunotherapy: CII.

[16] Kato K, Inagaki T, Shibagaki H, Yamauchi R, Okuda K, Sano T (1983). Structural analysis of the β-D-glucan extracted with aqueous zinc chloride from the fruit body of Grifola frondosa. Carbohydrate Research.

[17] Zhang J, Liu D, Wen C, Liu J, Xu X, Liu G (2022). New light on Grifola frondosa polysaccharides as biological response modifiers. Trends in Food Science & Technology.

[18] Xiao C, Jiao C, Xie Y, Ye L, Li Q, Wu Q (2021). Grifola frondosa GF5000 improves insulin resistance by modulation the composition of gut microbiota in diabetic rats. Journal of Functional Foods.

[19] Inoue A, Kodama N, Nanba H (2002). Effect of Maitake (Grifola frondosa) D-Fraction on the Control of the T Lymph Node Th-1/Th-2 Proportion. Biological & Pharmaceutical Bulletin.

[20] Kodama N, Komuta K, Nanba H (2003). Effect of Maitake (Grifola frondosa) D-Fraction on the activation of NK cells in cancer patients. Journal of Medicinal Food.

[21] Zhang Y, Mills GL, Nair MG (2002). Cyclooxygenase inhibitory and antioxidant compounds from the mycelia of the edible mushroom Grifola frondosa. J Agric Food Chem.

[22] Pulaski BA, Ostrand-Rosenberg S (2001). Mouse 4T1 breast tumor model. Current protocols in immunology.

[23] Schrörs B, Boegel S, Albrecht C, Bukur T, Bukur V, Holtsträter C (2020). Multi-Omics Characterization of the 4T1 Murine Mammary Gland Tumor Model. Front Oncol.

[24] Hawila E, Razon H, Wildbaum G, Blattner C, Sapir Y, Shaked Y (2017). CCR5 Directs the Mobilization of CD11b+Gr1+Ly6Clow Polymorphonuclear Myeloid Cells from the Bone Marrow to the Blood to Support Tumor Development. Cell Reports.

[25] Wu QP, Xie YZ, Deng Z, Li XM, Yang W, Jiao CW (2012). Ergosterol peroxide isolated from Ganoderma lucidum abolishes microRNA miR-378-mediated tumor cells on chemoresistance. PloS one.

[26] LaPierre DP, Lee DY, Li SZ, Xie YZ, Zhong L, Sheng W (2007). The ability of versican to simultaneously cause apoptotic resistance and sensitivity. Cancer research.

[27] Tian HX, Mei J, Cao L, Song J, Rong D, Fang M Disruption of Iron Homeostasis to Induce Ferroptosis with Albumin-Encapsulated Pt(IV) Nanodrug for the Treatment of Non-Small Cell Lung Cancer. Small (Weinheim an der Bergstrasse, Germany). 2023: e2206688.

[28] Xu G, Zhong Y, Munir S, Yang BB, Tsang BK, Peng C (2004). Nodal induces apoptosis and inhibits proliferation in human epithelial ovarian cancer cells via activin receptor-like kinase 7. The Journal of clinical endocrinology and metabolism.

[29] Li X, Sdiri M, Peng J, Xie Y, Yang BBJIJoBS Identification and characterization of chemical components in the bioactive fractions of Cynomorium coccineum that possess anticancer activity. 2020; 16: 61-73.

[30] Li X, Xie Y, Peng J, Hu H, Wu Q, Yang BB (2019). Ganoderiol F purified from Ganoderma leucocontextum retards cell cycle progression by inhibiting CDK4/CDK6. Cell Cycle.

[31] Xu J, Bai XH, Lodyga M, Han B, Xiao H, Keshavjee S (2007). XB130, a novel adaptor protein for signal transduction. The Journal of biological chemistry.

[32] Rutnam ZJ, Yang BB (2012). The non-coding 3' UTR of CD44 induces metastasis by regulating extracellular matrix functions. Journal of cell science.

[33] Rutnam ZJ, Du WW, Yang W, Yang X, Yang BB (2014). The pseudogene TUSC2P promotes TUSC2 function by binding multiple microRNAs. Nature Communications.

[34] Li X, Sdiri M, Peng J, Xie Y, Yang BB (2020). Identification and characterization of chemical components in the bioactive fractions of Cynomorium coccineum that possess anticancer activity. International Journal of Biological Sciences.

[35] Bronte V, Brandau S, Chen SH, Colombo MP, Frey AB, Greten TF (2016). Recommendations for myeloid-derived suppressor cell nomenclature and characterization standards. Nature Communications.

[36] Gabrilovich DI, Ostrand-Rosenberg S, Bronte V (2012). Coordinated regulation of myeloid cells by tumours. Nature Reviews Immunology.

[37] Yu J, Du W, Yan F, Wang Y, Li H, Cao S (2013). Myeloid-derived suppressor cells suppress antitumor immune responses through IDO expression and correlate with lymph node metastasis in patients with breast cancer. Journal of immunology (Baltimore, Md: 1950).

[38] Casbon AJ, Reynaud D, Park C, Khuc E, Gan DD, Schepers K (2015). Invasive breast cancer reprograms early myeloid differentiation in the bone marrow to generate immunosuppressive neutrophils. Proceedings of the National Academy of Sciences of the United States of America.

[39] Turbitt WJ, Xu Y, Sosnoski DM, Collins SD, Meng H, Mastro AM (2019). Physical Activity Plus Energy Restriction Prevents 4T1.2 Mammary Tumor Progression, MDSC Accumulation, and an Immunosuppressive Tumor Microenvironment. Cancer Prevention Research.

[40] Li BH, Jiang W, Zhang S, Huang N, Li ZF (2020). The spleen contributes to the increase in PMN-MDSCs in orthotopic H22 hepatoma mice. Molecular Immunology.

[41] Pulaski BA, Ostrand-Rosenberg S (2001). Mouse 4T1 Breast Tumor Model. Current Protocols in Immunology.

[42] Subramanian A, Tamayo P, Mootha VK, Mukherjee S, Ebert BL, Gillette MA (2005). Gene set enrichment analysis: A knowledge-based approach for interpreting genome-wide expression profiles. Proceedings of the National Academy of Sciences of the United States of America.

[43] Liberzon A, Birger C, Thorvaldsdóttir H, Ghandi M, Mesirov Jill P, Tamayo P (2015). The Molecular Signatures Database Hallmark Gene Set Collection. Cell Systems.

[44] Feng M, Jiang W, Kim BYS, Zhang CC, Fu Y-X, Weissman IL (2019). Phagocytosis checkpoints as new targets for cancer immunotherapy. Nature Reviews Cancer.

[45] Wu L, Mao L, Liu J-F, Chen L, Yu G-T, Yang L-L (2019). Blockade of TIGIT/CD155 Signaling Reverses T-cell Exhaustion and Enhances Antitumor Capability in Head and Neck Squamous Cell Carcinoma. Cancer Immunology Research.

[46] Robert C (2020). A decade of immune-checkpoint inhibitors in cancer therapy. Nature Communications.

[47] Broz Miranda L, Binnewies M, Boldajipour B, Nelson Amanda E, Pollack Joshua L, Erle David J (2014). Dissecting the Tumor Myeloid Compartment Reveals Rare Activating Antigen-Presenting Cells Critical for T Cell Immunity. Cancer Cell.

[48] Liu C, Qiang J, Deng Q, Xia J, Deng L, Zhou L (2021). ALDH1A1 Activity in Tumor-Initiating Cells Remodels Myeloid-Derived Suppressor Cells to Promote Breast Cancer Progression. Cancer research.

[49] Yoshino S, Tabata T, Hazama S, Iizuka N, Yamamoto K, Hirayama M (2000). Immunoregulatory effects of the antitumor polysaccharide lentinan on Th1/Th2 balance in patients with digestive cancers. Anticancer Res.

[50] Zhu X-L, Chen A-F, Lin Z-B (2007). Ganoderma lucidum polysaccharides enhance the function of immunological effector cells in immunosuppressed mice. Journal of Ethnopharmacology.

[51] Wasser SP (2017). Medicinal Mushrooms in Human Clinical Studies. Part I. Anticancer, Oncoimmunological, and Immunomodulatory Activities: A Review. International journal of medicinal mushrooms.

[52] Du J, Wang R, Zhang W, Zhang C, Li X, Shi X (2017). A polysaccharide derived from Lentinus edodes impairs the immunosuppressive function of myeloid-derived suppressor cells via the p38 pathways. RSC Adv.

[53] Wang Y, Fan X, Wu X (2020). Ganoderma lucidum polysaccharide (GLP) enhances antitumor immune response by regulating differentiation and inhibition of MDSCs via a CARD9-NF-κB-IDO pathway. Bioscience Reports.

[54] Wu H, Tao N, Liu X, Li X, Tang J, Ma C (2012). Polysaccharide from Lentinus edodes inhibits the immunosuppressive function of myeloid-derived suppressor cells. PLoS One.

[55] Du J, Wang R, Zhang W, Zhang C, Li X, Shi X (2017). A polysaccharide derived from Lentinus edodes impairs the immunosuppressive function of myeloid-derived suppressor cells via the p38 pathways. RSC Advances.

[56] Zhang Y, Li S, Wang X, Zhang L, Cheung PCK (2011). Advances in lentinan: Isolation, structure, chain conformation and bioactivities. Food Hydrocolloids.

[57] He X, Wang X, Fang J, Chang Y, Ning N, Guo H (2017). Polysaccharides in Grifola frondosa mushroom and their health promoting properties: A review. International Journal of Biological Macromolecules.

[58] Johnston Robert J, Comps-Agrar L, Hackney J, Yu X, Huseni M, Yang Y (2014). The Immunoreceptor TIGIT Regulates Antitumor and Antiviral CD8+ T Cell Effector Function. Cancer Cell.

[59] Harjunpää H, Guillerey C (2019). TIGIT as an emerging immune checkpoint. Clinical and Experimental Immunology.

[60] Gabrilovich DI, Nagaraj S (2009). Myeloid-derived suppressor cells as regulators of the immune system. Nature Reviews Immunology.

[61] Cortez-Retamozo V, Etzrodt M, Newton A, Rauch PJ, Chudnovskiy A, Berger C (2012). Origins of tumor-associated macrophages and neutrophils. Proceedings of the National Academy of Sciences.

[62] Melero-Jerez C, Alonso-Gómez A, Moñivas E, Lebrón-Galán R, Machín-Díaz I, de Castro F (2020). The proportion of myeloid-derived suppressor cells in the spleen is related to the severity of the clinical course and tissue damage extent in a murine model of multiple sclerosis. Neurobiology of Disease.

