# Supplementary material for: Polysaccharide isolated from Grifola frondosa eliminates myeloid-derived suppressor cells and inhibits tumor growth by enhancing T cells responses
Source: Int J Biol Sci. 2024 Jan 1;20(2):664–79. doi: 10.7150/ijbs.85276 (PMC10758108; doi:10.7150/ijbs.85276)
Supplement: Supplementary file 1 — Supplementary figures. [file ijbsv20p0664s1.pdf]

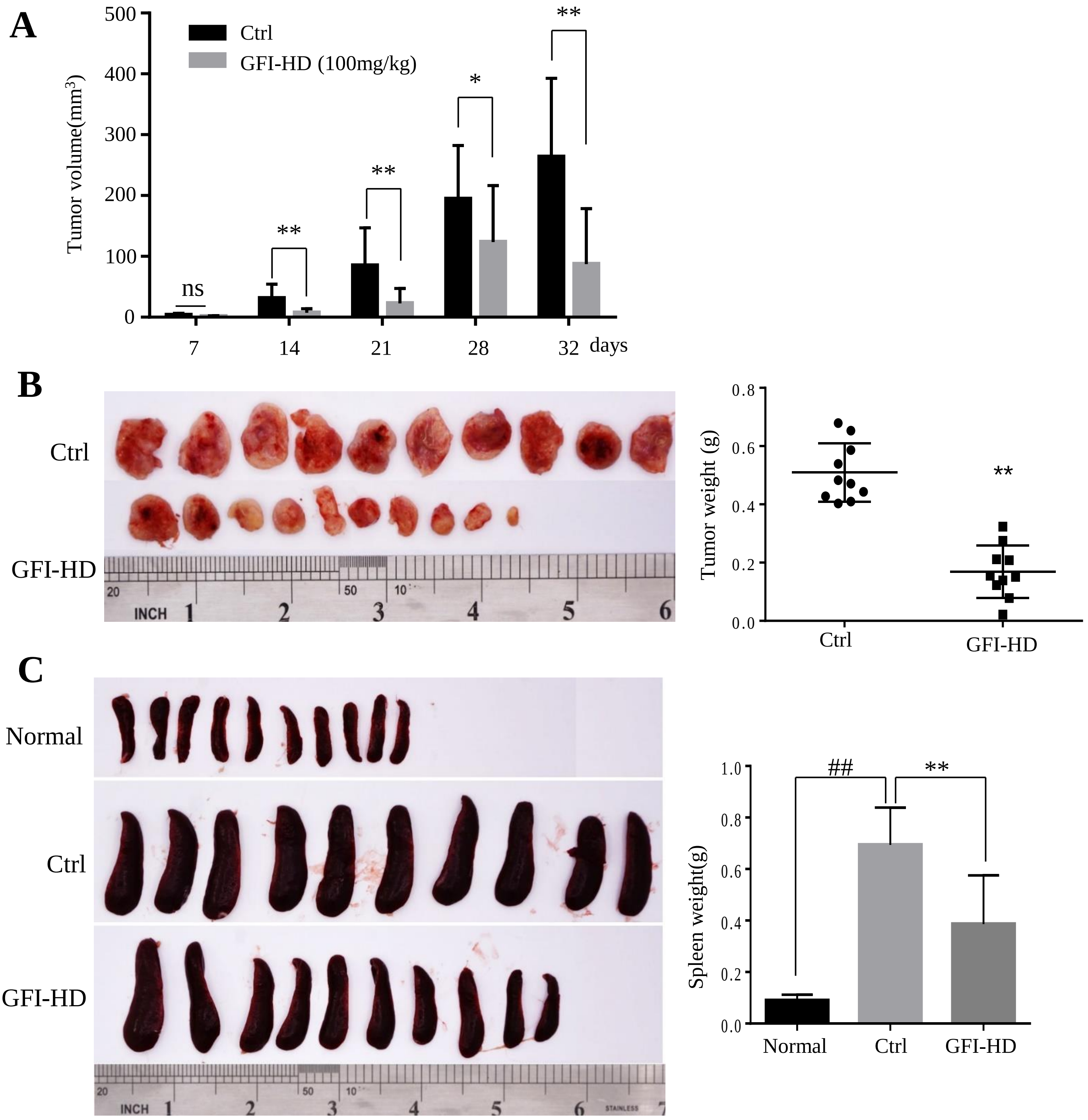

Supplementary Figure S1. Inhibition of tumor growth.

The second day after 4T1 cells implantation into mice, the mice were given intraperitoneal injections of GFI (100 mg/kg/per mouse) or 0.9% saline once every other day up to 32 days.

(A) The tumor volume of mice in each group of the 4T1 model were measured and calculated at the indicated time points (n = 10).

(B) Left: Representative photos of tumor; Right: the tumor weight of mice were measured and calculated at the end of assay (n = 10).

(C) Left: Representative photos of spleen; Right: the spleen weight of mice were measured and calculated at the end of assay (n = 10). *p<0.05; *p<0.01; #p<0.05; ##p<0.01. Error bars, SD.

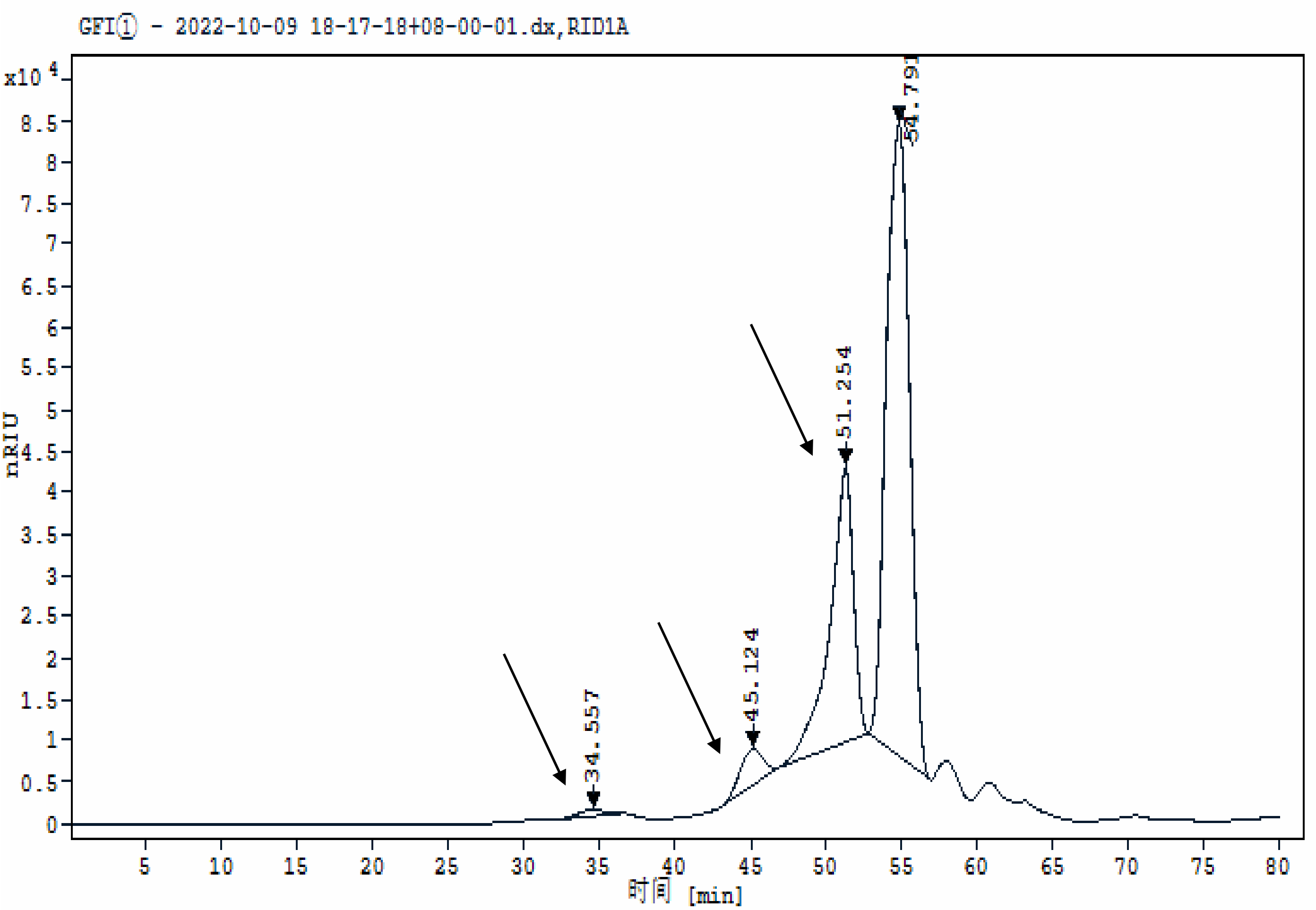

Supplementary Figure S2. Purification of bioactive components.

Chromatogram of the polysaccharides from the fruit bodies of *Grifola frondosa* sample

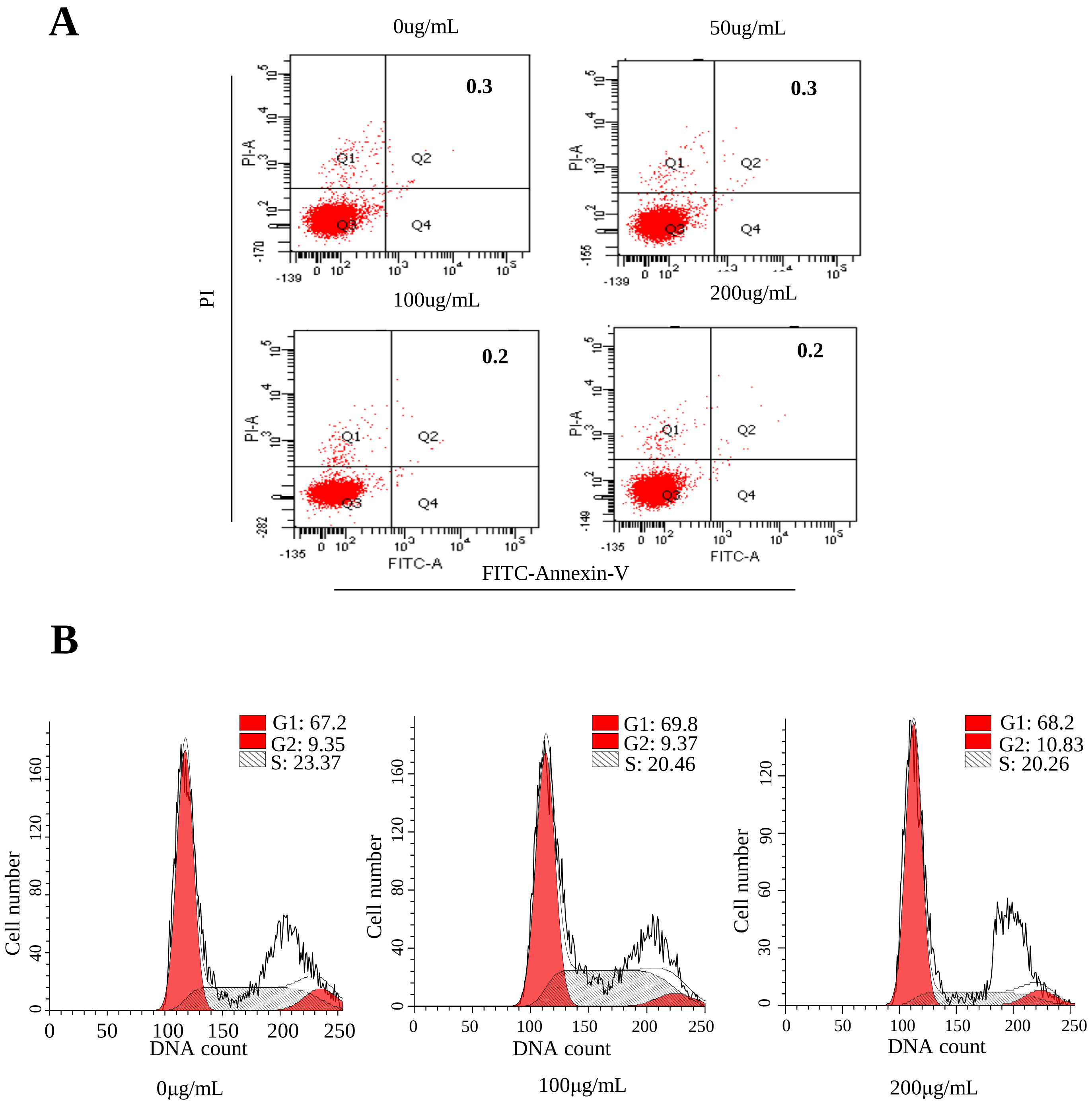

Supplementary Figure S3. Tumor cell flow cytometry.

(A) Representative FACS plots of cell apoptosis.

(B) Histogram of cell cycle.

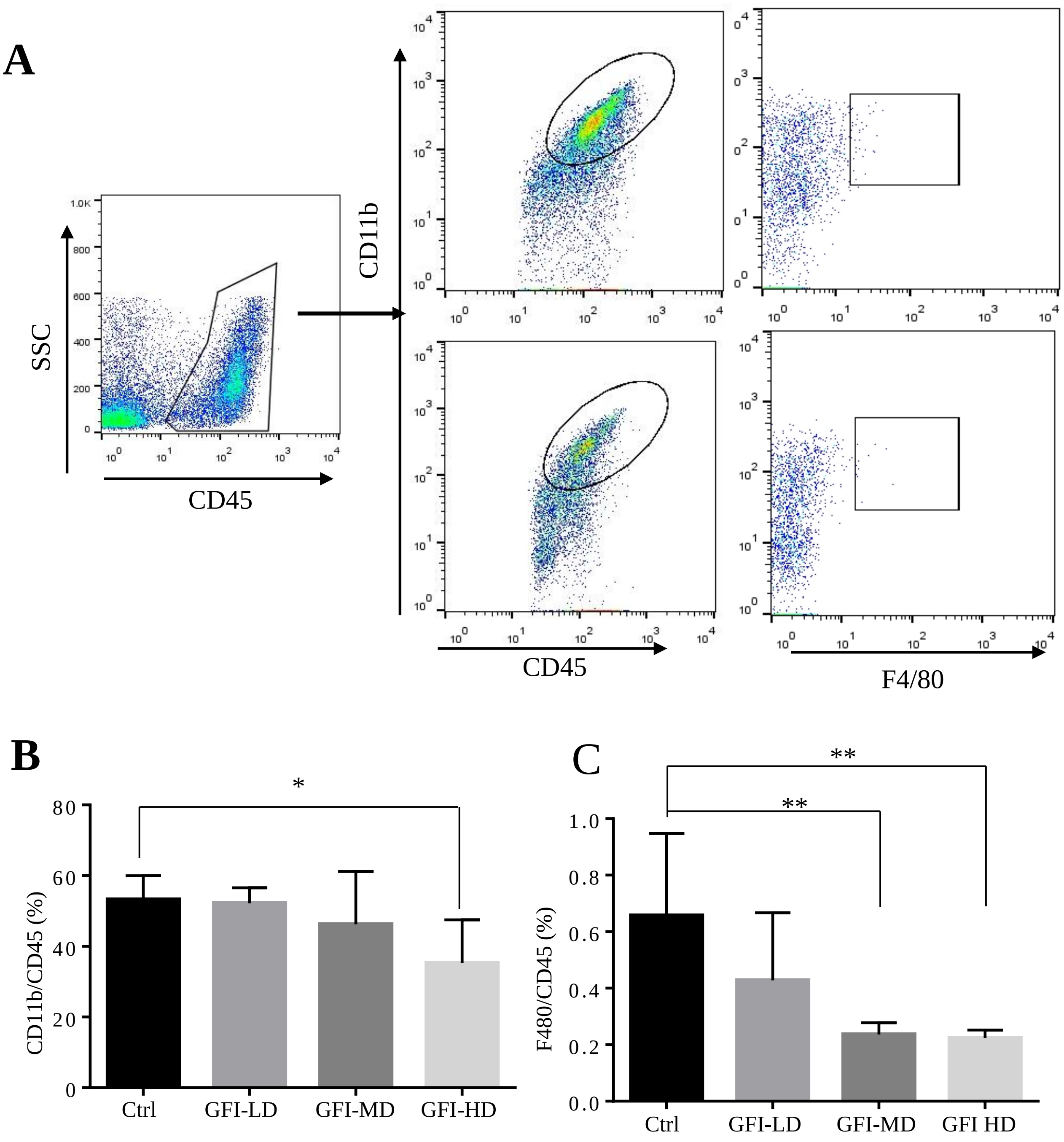

Supplementary Figure S4. Tumor cell flow cytometry.

(A) Representative FACS plots illustrate the gating to assess F4/80 cells.

(B) The alteration of CD11b$^+$ myeloid cells in the tumor from the control or GFI-treated 4T1 murine model using FACS analysis.

(C) The alteration of CD11b$^+$ F4/80$^+$ TAMs in the tumor from the control or GFI-treated 4T1 murine model using FACS analysis. *$p<0.05$; *$p<0.01$. Error bars, SD.

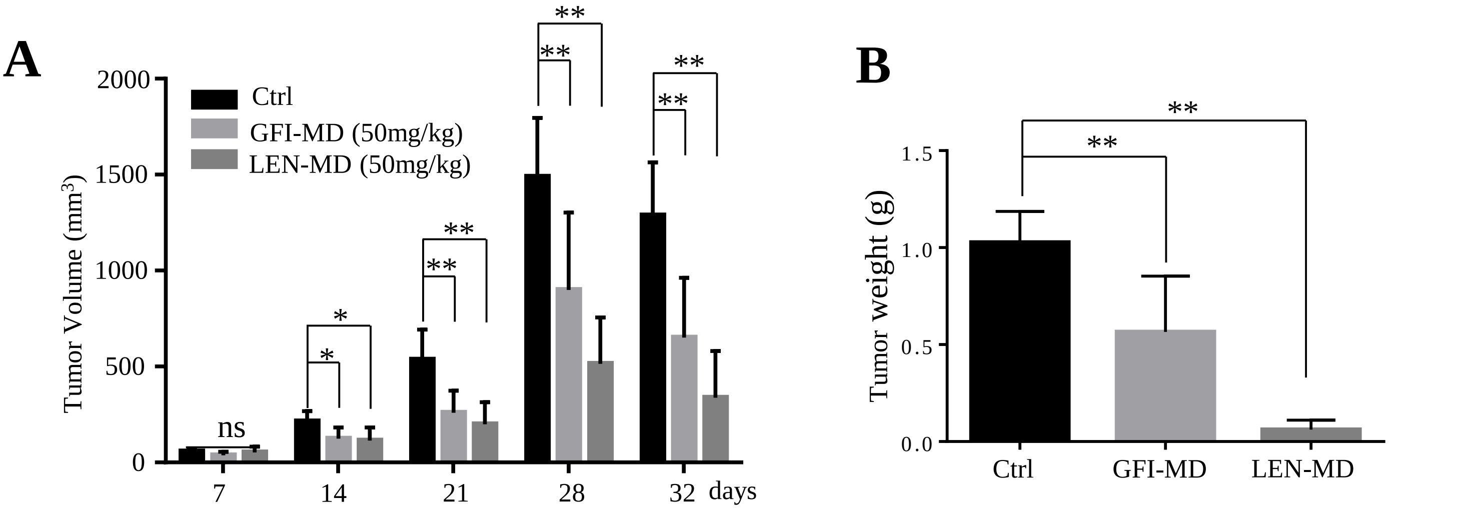

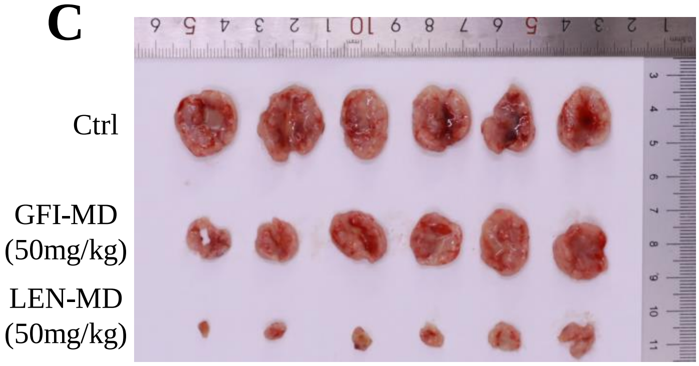

Sample name	Tumor weight (means ± SD, g)	Tumor-inhibition rate(means ± SEM, %)
Ctrl	1.026±0.16	--
GFI-MD (50mg/kg)	0.566±0.287**	44.83±11.57
LEN-HD (50mg/kg)	0.061±0.047****	93.99±1.90

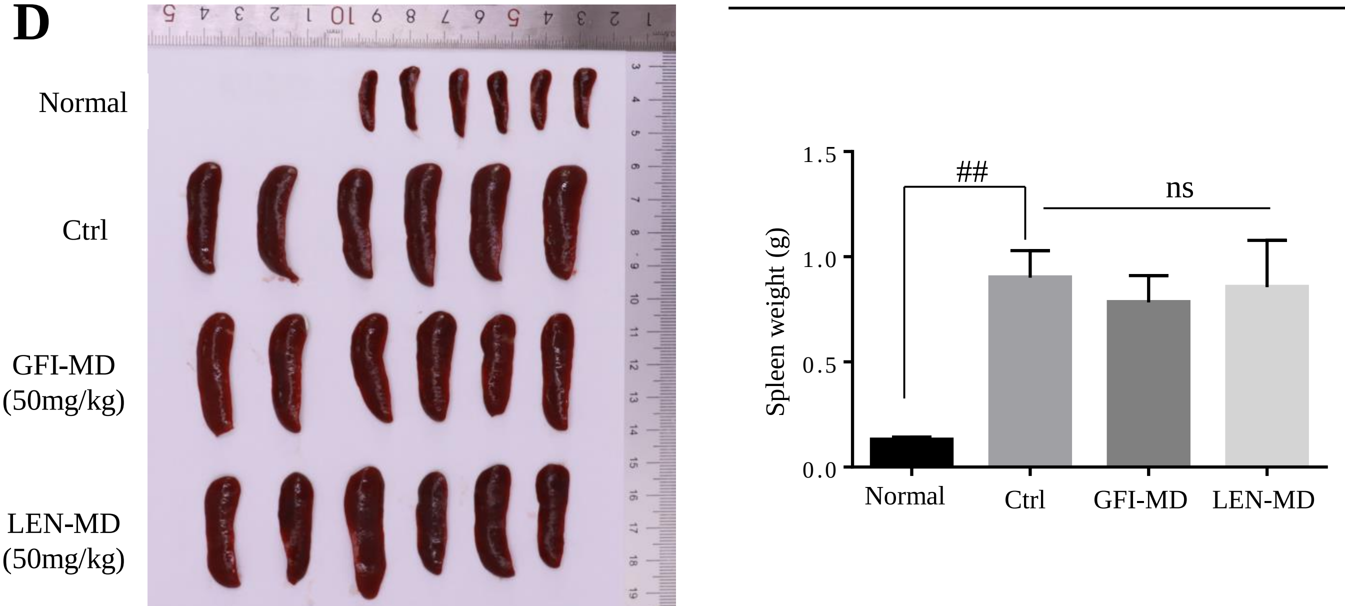

Supplementary Figure S5. Compared the antitumor effect of polysaccharide from *G. frondosa* and *L. edodes in vivo*

At one week tumor inoculation after 4T1 cells ($0.5x10^5$cells/mouse) implantation into mice, the mice were given intraperitoneal injections of GFI (50mg/kg, GFI-MD), or lentinan (50mg/kg, LEN-MD) once every other day up to 35-day, with 100μL. Control group were given intraperitoneal injections of 0.9% saline.

(A) The tumor volumes of the mice were measured and calculated weekly (n = 6).

(B) The body weight of mice was weighed and calculated weekly (n = 6).

(C) Left: representative photos showing the effect of GFI on tumor growth inhibition in the 4T1 murine model (n = 6); Right: shown tumor weight and tumor-inhibition rate (means ± SEM) (n = 6).

(D) Left: representative photos of spleen in each group (n = 6); Right: shown spleen weight (n = 6). *p<0.05; **p<0.01. Error bars (no specifically indicated), SD.

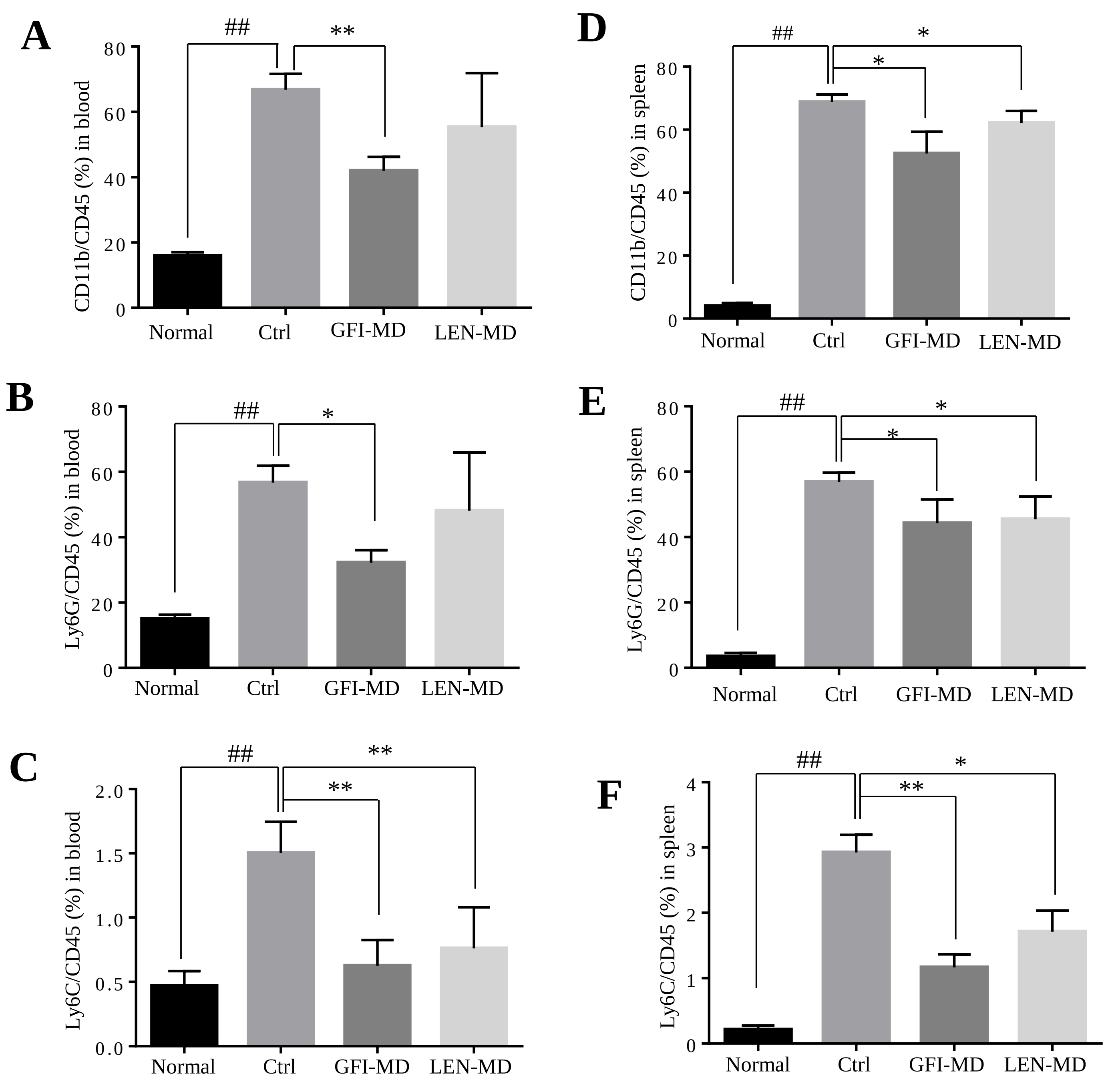

Supplementary Figure S6. Polysaccharide from *G. frondosa* and *L. edodes* suppresses the enrichment of MDSCs in the peripheral blood and spleen.

After the mice were sacrificed, MDSCs were analyzed with cell surface markers ($CD45^{+}CD11b^{+}$) in peripheral blood and spleen from the control, or GFI treatment group, or LEN treatment group in 4T1 murine model using flow cytometry (n = 4).

(A) GFI treatment significantly decreased $CD11b^{+}$MDSCs enrichment in the peripheral blood as observed by using flow cytometry analysis; LEN treatment also reduced the percentage of $CD11b^{+}$MDSCs in the peripheral blood, but not significantly.

(B) Flow cytometry analyzed the density of PMN-MDSCs and M-MDSCs in the peripheral blood from the control or GFI-treated 4T1 murine model, staining with CD45, CD11b, Ly6G and Ly6C antibodies. GFI-MD treatment significantly decreased the percentage of PMN-MDSCs, but LEN-MD treatment did not.

(C) GFI-MD and LEN-MD treatment significantly decreased the percentage of M-MDSCs in the peripheral blood of 4T1 murine model.

(D) GFI treatment and LEN treatment significantly decreased $CD11b^{+}$MDSCs enrichment in the spleen of 4T1 murine model.

(E) GFI-MD and LEN-MD treatment significantly reduced the percentage of PMN-MDSCs in the spleen of 4T1 murine model.

(F) GFI-MD and LEN-MD treatment significantly decreased the percentage of M-MDSCs in the spleen of 4T1 murine model.

*$p<0.05$; *$p<0.01$; #$p<0.05$; ##$p<0.01$; Error bars, SD.
