# Supplementary material for: Polysaccharide isolated from Grifola frondosa eliminates myeloid-derived suppressor cells and inhibits tumor growth by enhancing T cells responses
Source: Int J Biol Sci. 2024 Jan 1;20(2):664–79. doi: 10.7150/ijbs.85276 (PMC10758108; doi:10.7150/ijbs.85276)
Supplement: Supplementary file 2 — Supplementary tables. [file ijbsv20p0664s2.pdf]

Supplementary Table S1. Key Resources used in the study

REAGENT	SOURCE	IDENTIFIER
Antibodies		
Anti-mouse CD45- APC-eFluor 780 (30-F11)	eBioscience	47-0451-82
Anti-mouse CD11b-APC (M1/70)	eBioscience	11-0031-82
Anti-mouse CD3e- FITC (145-2C11)	eBioscience	17-0112-82
Anti-mouse Ly-6G/Ly-6C(GR-1)- PE-Cyanine7 (RB6-8C5)	eBioscience	25-5931-82
Anti-mouse Ly6G-FITC (1A8)	eBioscience	11-9668-82
Anti-mouse Ly6C- PE-Cyanine7 (HK1.4)	eBioscience	25-5932-82
Anti-mouse F4/80-PE (BM8)		12-4801-82
Anti-mouse CD4-APC (GK1.5)	eBioscience	17-0041-82
Anti-mouse CD8-PE (53-6.7)	eBioscience	12-0081-82
Anti-Mouse CD16/CD32 (Mouse BD Fc Block™)	BD	553141
Anti-mouse CD11b (EPR1344)	abcam	ab133357
Anti-mouse Ly6g(EPR22909-135)	abcam	ab238132
Anti-mouse CD3 (SP7)	abcam	ab16669
Anti-mouse CD4 (PR19514)	abcam	ab183685
Anti-mouse CD8α (EPR21769)	abcam	ab217344
Anti-mouse TIGIT(EPR26037-152)	abcam	ab300073
Anti-mouse Granzyme B (EPR22645-206)	abcam	ab255598
Ki67 (D3B5)	Cell signaling technology	12202
Goat anti-Rat IgG H&L (HRP)	abcam	ab6721
Chemical		
DNase, Type I	Sigma-Aldrich	D4527
Collagenase from Clostridium Histolyticum, Type IV	Sigma-Aldrich	C5138
Dispase II	Sigma-Aldrich	D4693
7-AAD	BD	559925

Supplementary Table S2. Molecular weight of polysaccharides from the fruit bodies of Grifola frondosa sample

Retention Time (min)	Mw(x10^4Da)
34.557	99.93
45.124	2.07
51.254	0.244

Supplementary Table S3. Sequences of primers used in the study

Gene	Primer sequence
mus Tigit	F: GGCCTCACCTTCCAGTCTC<br>R: ATTCTCCCCTTGTAAATCCCAC
mus GzmB	F: ACCCAGCAAGTCATCCCTA<br>R: GGCCTTACTCTTCAGCTTTA
mus GAPDH	F: GCCAAAAGGGTCATCATCTCCG<br>R: ATGAGCCCTTCCACAATGCC
